# Folding a Molecular Strand into a Trefoil Knot of Single Handedness with Co(II)/Co(III) Chaperones

**DOI:** 10.1021/jacs.4c05953

**Published:** 2024-07-26

**Authors:** Jiankang Zhong, Zhanhu Sun, Liang Zhang, George F. S. Whitehead, Iñigo J. Vitorica-Yrezabal, David A. Leigh

**Affiliations:** † School of Chemistry and Molecular Engineering, East China Normal University, 200062 Shanghai, China; ‡ Department of Chemistry, University of Manchester, Manchester M13 9PL, U.K.

## Abstract

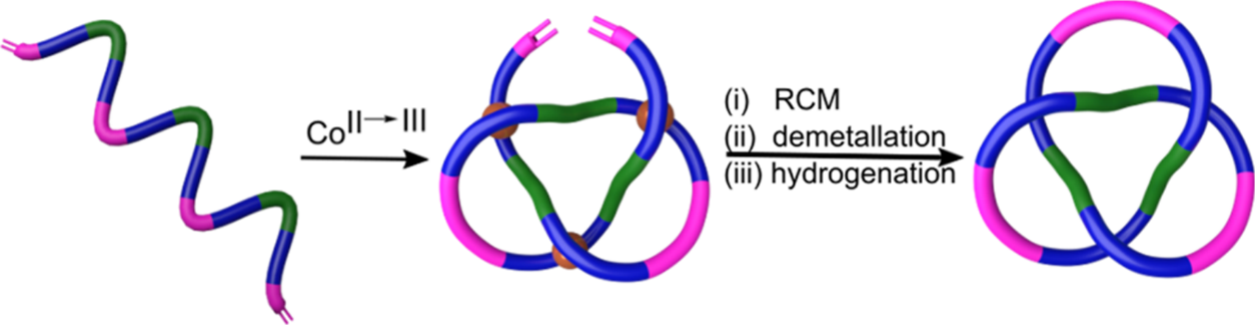

We report the synthesis of a right-handed (Δ-stereochemistry of strand crossings) trefoil knot from a single molecular strand containing three pyrazine-2,5-dicarboxamide units adjacent to point-chiral centers and six pyridine moieties. The oligomeric ligand strand folds into an overhand (open-trefoil) knot through the assistance of coordinatively dynamic Co(II) “chaperones” that drive the formation of a three-metal-ion circular helicate. The entangled structure is kinetically locked by oxidation to Co(III) and covalently captured by ring-closing olefin metathesis to generate a trefoil knot of single topological handedness. The stereochemistry of the strand crossings in the metal-coordinated overhand knot is governed by the stereochemistry of the point-chiral carbon centers in the ligand strand. The overhand and trefoil knots were characterized by NMR spectroscopy, mass spectrometry, and X-ray crystallography. Removal of the metal ions from the knot, followed by hydrogenation of the alkene, yielded the wholly organic trefoil knot. The metal-free knot and parent ligand were investigated by circular dichroism (CD) spectroscopy. The CD spectra indicate that the topological stereochemistry of the knot has a greater effect on the asymmetry of the chromophore environment than do the point-chiral centers of the strand.

## Introduction

Knots are fundamental elements of structure at both the macroscopic and molecular levels.^1^ Knots and entanglements are found in proteins,^2^ DNA,^3^ and, most recently, RNA,^4^ and also form randomly and spontaneously in any polymers of sufficient length and flexibility.^5^ However, synthetic access to well-defined molecular knots remains challenging^6^ and the elucidation of the effects on properties that knotting produces is correspondingly underexplored.^7^ The formation of protein knots is often promoted by molecular chaperones that assist the folding of a single strand into the requisite crossing pattern.^8,9^ In contrast, most synthetic molecular knots have been prepared by the high-symmetry assembly of multiple building blocks, often through the covalent capture of circular metal helicates.^10^ In an alternative approach,^11^ labile metal ions (Zn(II), Cu(II), Ln(III)) have been used as single- or two-ion templates to fold oligomeric ligands into precursors to molecular knots.^7n,12^ Here, we combine aspects of the two approaches, using coordinatively labile Co(II) ions to fold an oligomeric ligand strand into a high-symmetry circular helicate, which is then kinetically locked by oxidation of the metal ions to Co(III). Ring-closing olefin metathesis then covalently captures the entangled architecture to form a molecular knot. The structural asymmetry of point-chiral centers in the ligand strand results in the knot being of a single topological handedness.

**Scheme 1:**
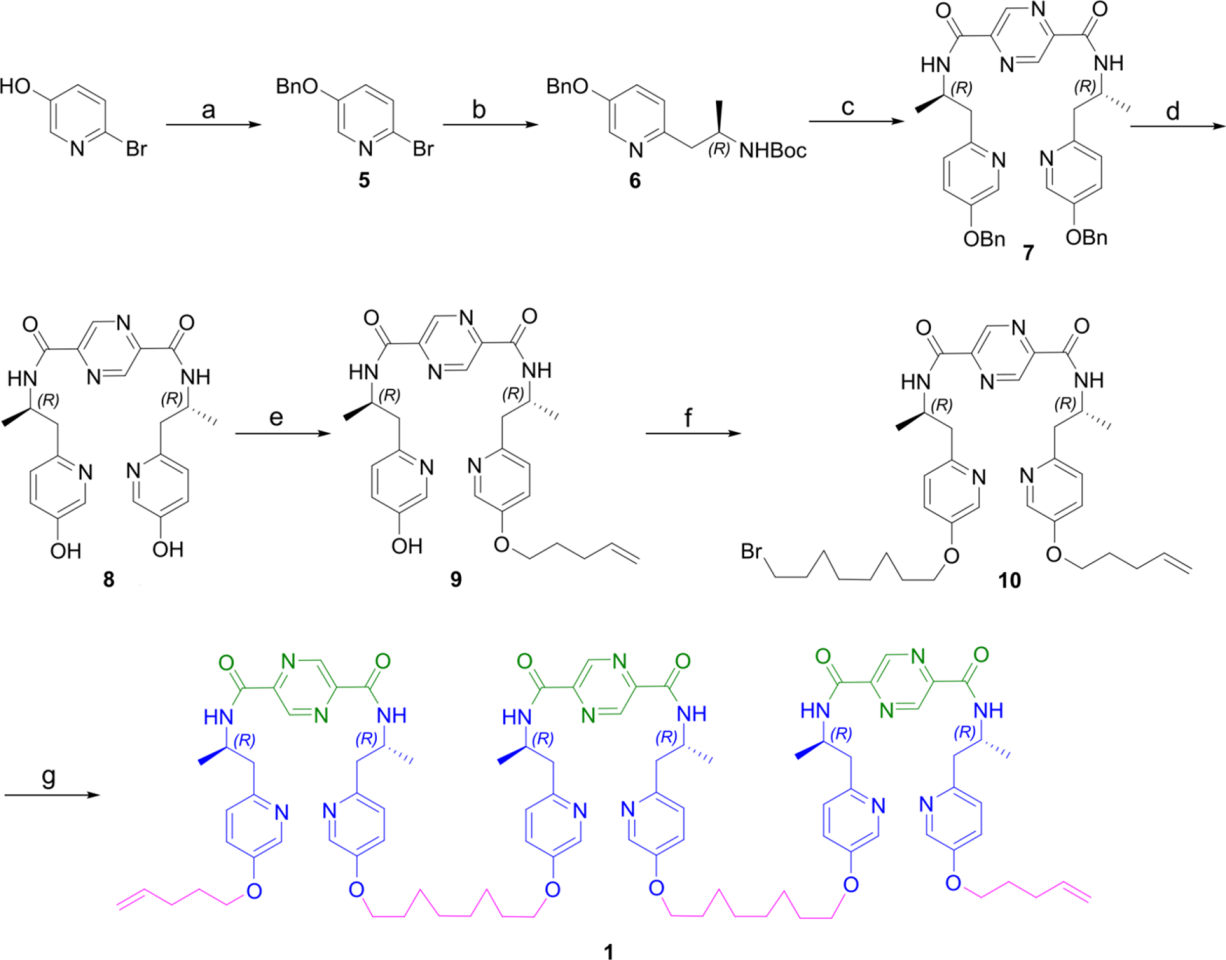
Synthesis of Ligand **1** Reagents and conditions: (a) benzyl bromide, K_2_CO_3_, DMF, 60 °C, 16 h, quant.; (b) Zn, I_2_, (*R*)-*tert*-butyl(1-iodopropan-2-yl)carbamate, Pd(PPh_3_)_2_Cl_2_, DMF, 0 to 45 °C, 48 h, 75%; (c) TFA, CH_2_Cl_2_, 0 °C to rt, 12 h, then pyrazine-2,5-dicarbonyl chloride, Et_3_N, CH_2_Cl_2_, 0 °C to rt, 4 h, 85%; (e) H_2_, Pd/C, MeOH, 45 °C, 2 h, quant.; (f) 5-bromo-1-pentene, K_2_CO_3_, DMF, 60 °C for 1 h, then rt, 3 d, 47%; (g) 1,8-dibromo-octane, K_2_CO_3_, DMF, 50 °C, 18 h, 87%; (h) **8**, K_2_CO_3_, DMF, 50 °C, 3 d, 65%.

We recently reported the two-step assembly of a homochiral trefoil knot *via* a trimeric circular helicate based on the chelation of bidentate chiral pyrazine-2,5-dicarboxamide ligands to Co(III) ions.^13^ We envisioned that linking the three pyrazine-diamide ligands together into a single stand **1** could lead to octahedral metal ions folding and entangling the strand into an overhand (*i.e*., not fully cyclized) trefoil knot (Scheme 1). However, Co(III) has very slow coordination kinetics which could mean that “mistakes” in terms of which bidentate unit of the oligomeric ligand binds to which metal ion coordination site would be slow to correct under thermodynamic control. Accordingly, we investigated a protocol in which the ligand strand is treated with Co(II), an octahedral metal ion that undergoes fast coordination kinetics, and once the most energetically favorable assembly is established, it is then oxidized *in situ* to a kinetically inert Co(III) coordination complex.

## Results and Discussion

The synthesis of tritopic molecular strand **1** was carried out as outlined in Scheme 1 from commercially available 2-bromo-5-hydroxypyridine. Benzyl protection followed by palladium-catalyzed Negishi cross-coupling with (*R*)-*tert*-butyl(1-iodopropan-2-yl)carbamate afforded **5** in 75% yield. Subsequent deprotection of the *N*-Boc group by trifluoroacetic acid and amidation with pyrazine-2,5-dicarbonyl chloride yielded **7** in 85% yield.^13^ Removal of the benzyl groups by hydrogenation and a subsequent desymmetrization with 5-bromo-1-pentene provided intermediate compound **9**. The linker (spacer length suggested by molecular modeling) was installed *via* Williamson ether synthesis to generate **10**, which was subjected to another 2-fold Williamson ether synthesis reaction with **8** to deliver the target oligomeric ligand strand **1** in 57% yield over two steps.^12b,12c^

**Scheme 2:**
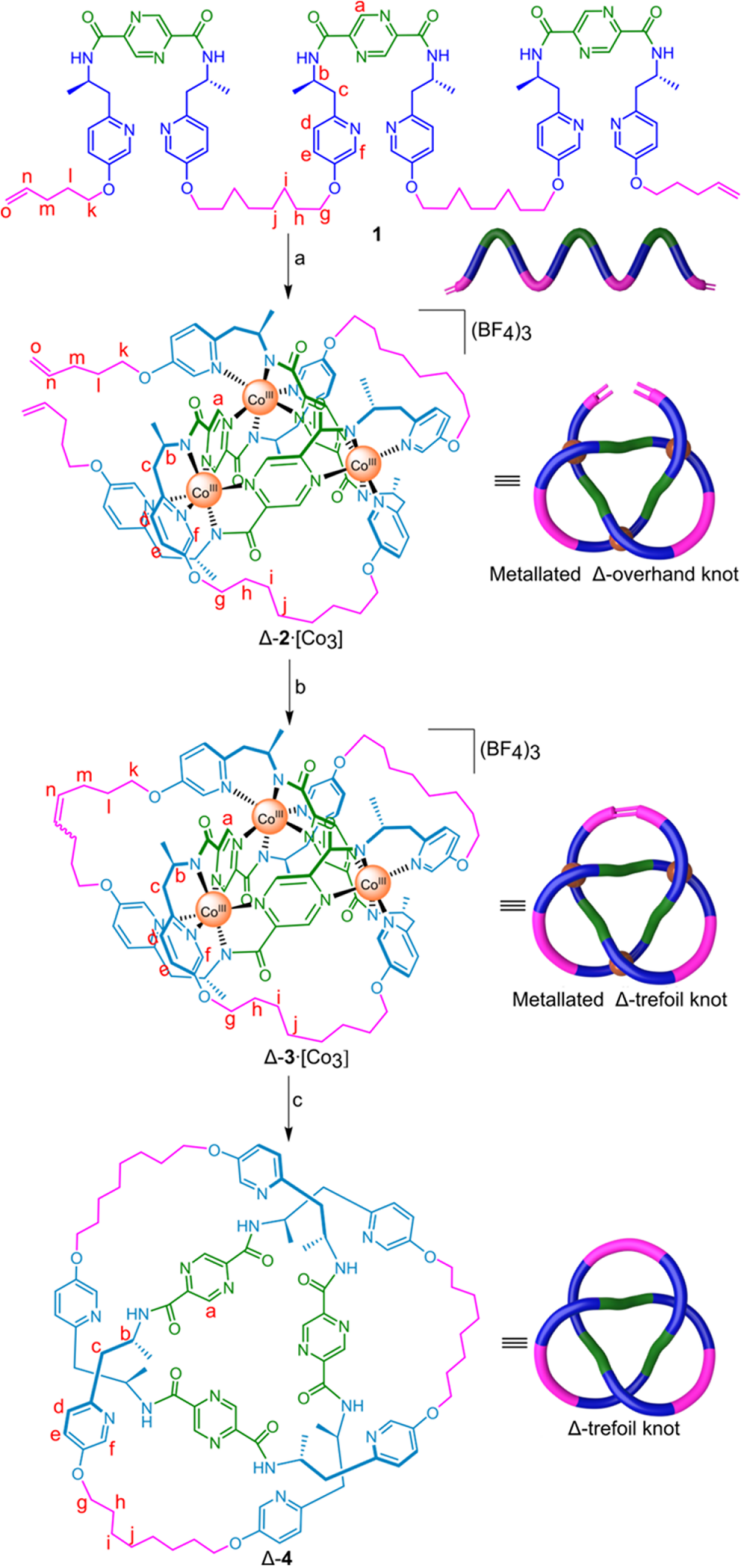
Synthesis of Right-Handed Trefoil Knot Δ-**4** Reagents and conditions: (a) Co(BF_4_)_2_·6H_2_O, Et_3_N, acetonitrile, 85 °C, 36 h, then O_2_, 85 °C, 3 days, 85%; (b) Hoveyda-Grubbs second-generation catalyst, nitromethane/1,2-dichloroethane (1:3), 60 °C, 48 h, 99%; (c) Zn, CH_3_COOH/MeOH (1/1), rt, 2 h, then H_2_, Pd/C, CH_2_Cl_2_/MeOH (1/1), 40 °C, 24 h, 35%.

**Figure 1:**
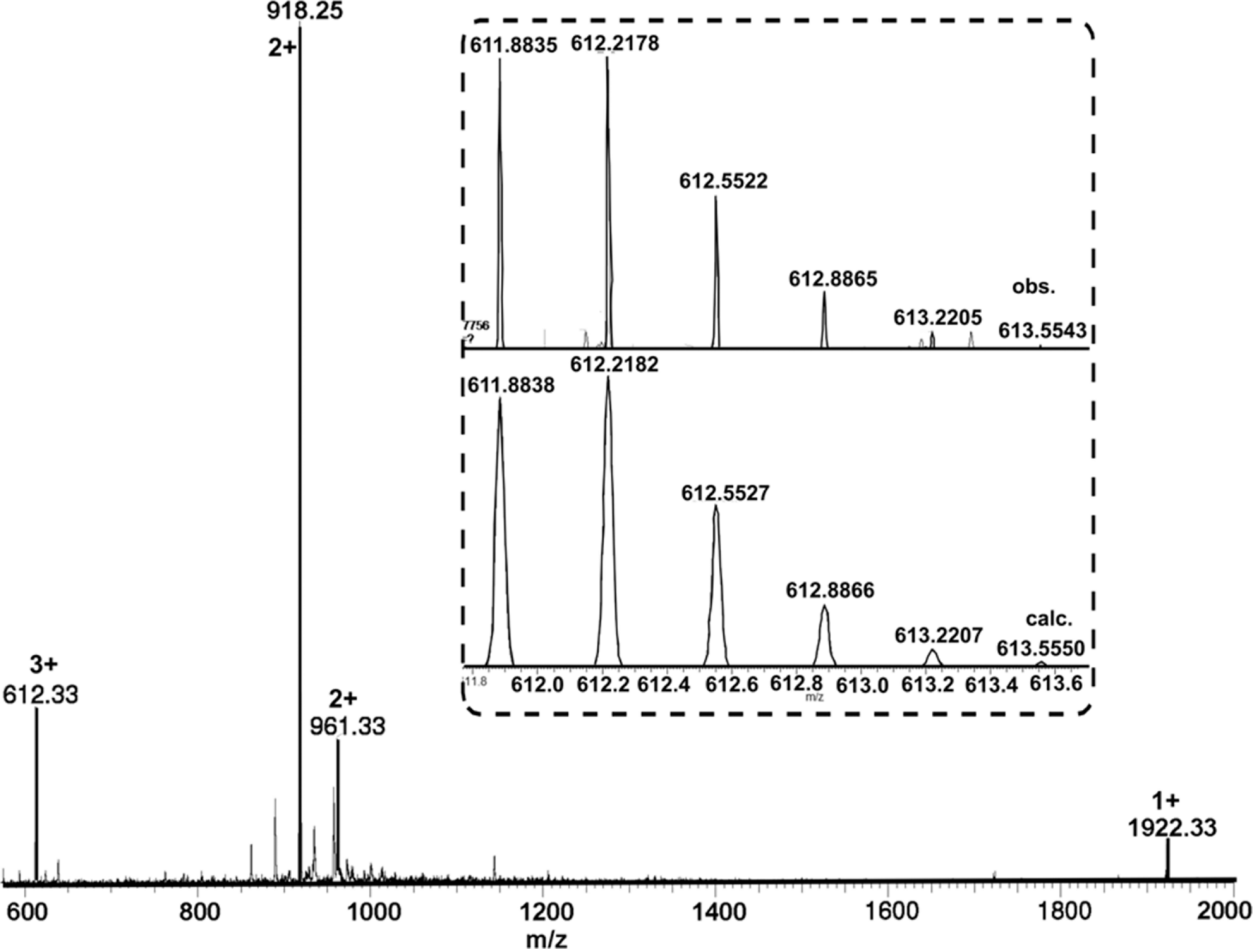
Low-resolution ESI-MS (positive mode) of overhand knot complex Δ-**2**·[Co_3_]. Inset: isotopic distribution of the [M-3BF_4_]^3+^ signal from high-resolution ESI-MS.

We investigated the folding process of **1** using a Co(II) salt, which was then oxidized *in situ* (Scheme 2, step a). The reaction of **1**, Co(BF_4_)_2_·6H_2_O, and Et_3_N in a 1:3.3:9 stoichiometry at 85 °C in acetonitrile under anaerobic conditions led to an immediate color change from light yellow to red-brown, indicating the formation of Co(II) complexes. The reaction was stirred at 85 °C for 36 h to allow exchange to occur to maximize the formation of the most thermodynamically stable form of [**1**-6H]·[Co(II)_3_]. Attempted oxidation of Co(II) to Co(III) was then carried out by bubbling oxygen through the reaction mixture at 85 °C for a further 3 days. Precipitation with diethyl ether afforded complex Δ-**2**·[Co_3_] in 85% yield (Scheme 2, step a). Electrospray ionization mass spectrometry (ESI-MS) shows a mixture of Co(III) and Co(II) ions in the ligand–metal complex (*m*/*z***2**·[Co(III)_3_]^3+^ 612.33, **2**·[Co(III)_2_Co(II)]^2+^ 918.25, **2**·[Co(III)_3_][BF_4_]^2+^ 961.33, **2**·[Co(III)_2_Co(II)][BF_4_]^+^ 1922.33 (Figure 1)). Furthermore, signals occurred at *m*/*z* = 612. 2173 and 918.3267 in high-resolution electrospray ionization mass spectrometry (HRESI-MS) are in good agreement with theoretical distributions of **2**·[Co(III)_3_]^3+^ and **2**·[Co(III)_2_Co(II)]^2+^, respectively (Figure 1, inset, Supporting Information Figure S2). We cannot confidently assign the Co(III)/Co(II) ratio from mass spectrometry, as the complexes may give rise to different signal intensities under electrospray conditions.

**Figure 2:**
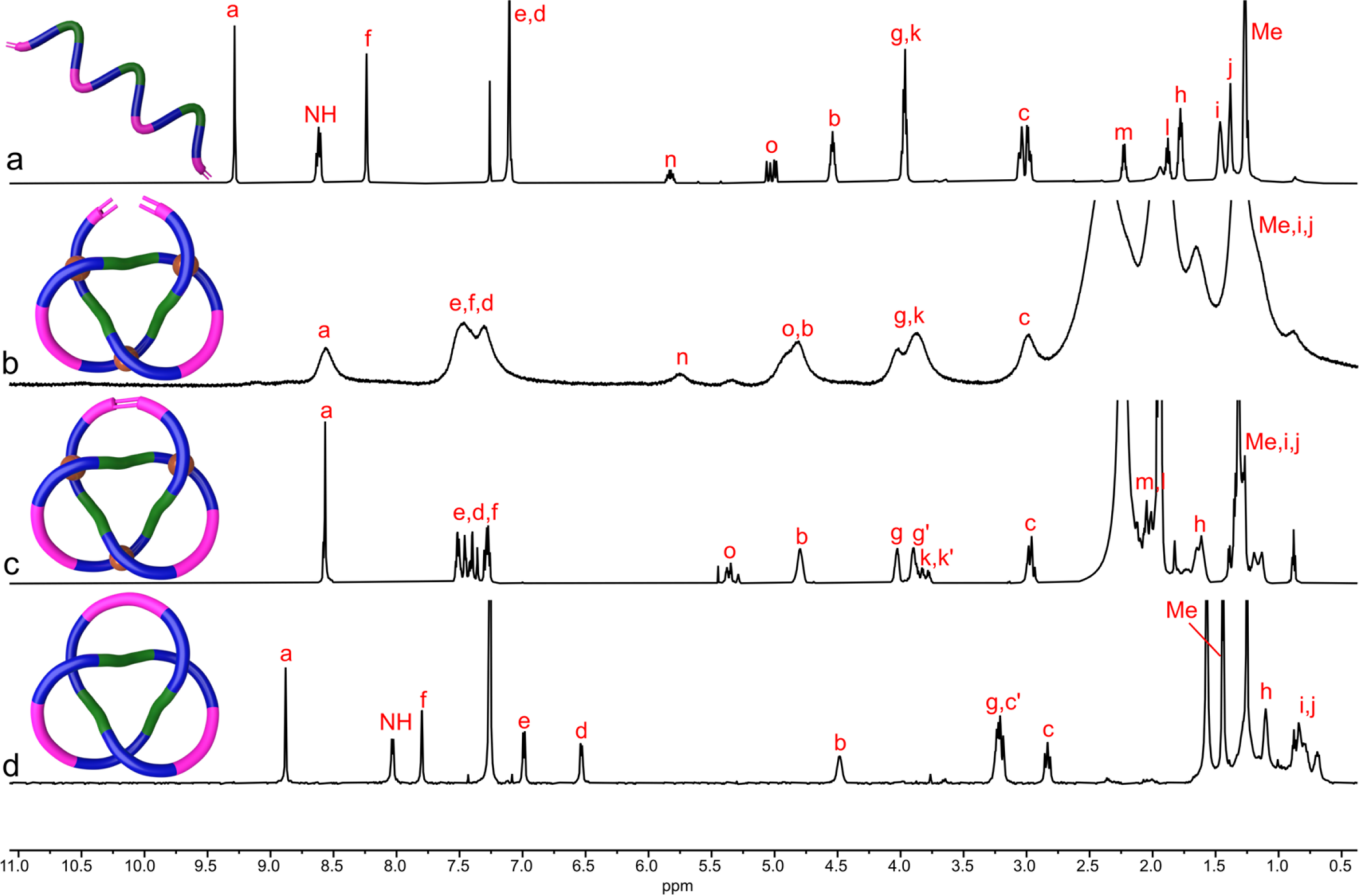
^1^H NMR Spectra (600 MHz, 298 K) of (a) amide ligand strand **1** (in CDCl_3_); (b) overhand knot Δ-**2**·[Co_3_] (in CD_3_CN); (c) metalated trefoil knot Δ-**3**·[Co_3_] (in CD_3_CN); (d) metal-free trefoil knot Δ-**4** (in CDCl_3_). Signal labels correspond to the atom labels in Scheme 2.

The ^1^H NMR spectrum of Δ-**2**·[Co_3_] is broad (Figure 2b), consistent with the residual paramagnetic high-spin Co(II) centers indicated by mass spectrometry. The absence of signals for amide protons and the substantial chemical shifts of the pyridine and pyrazine protons with respect to those of the free ligand indicate that all of the nitrogen atoms of the ligand strand are coordinated to metal ions. ^1^H Diffusion ordered spectroscopy (DOSY) shows that all of the protons of the complex have the same diffusion coefficient, indicating a single species present in solution (Supporting Information Figure S24).

**Figure 3:**
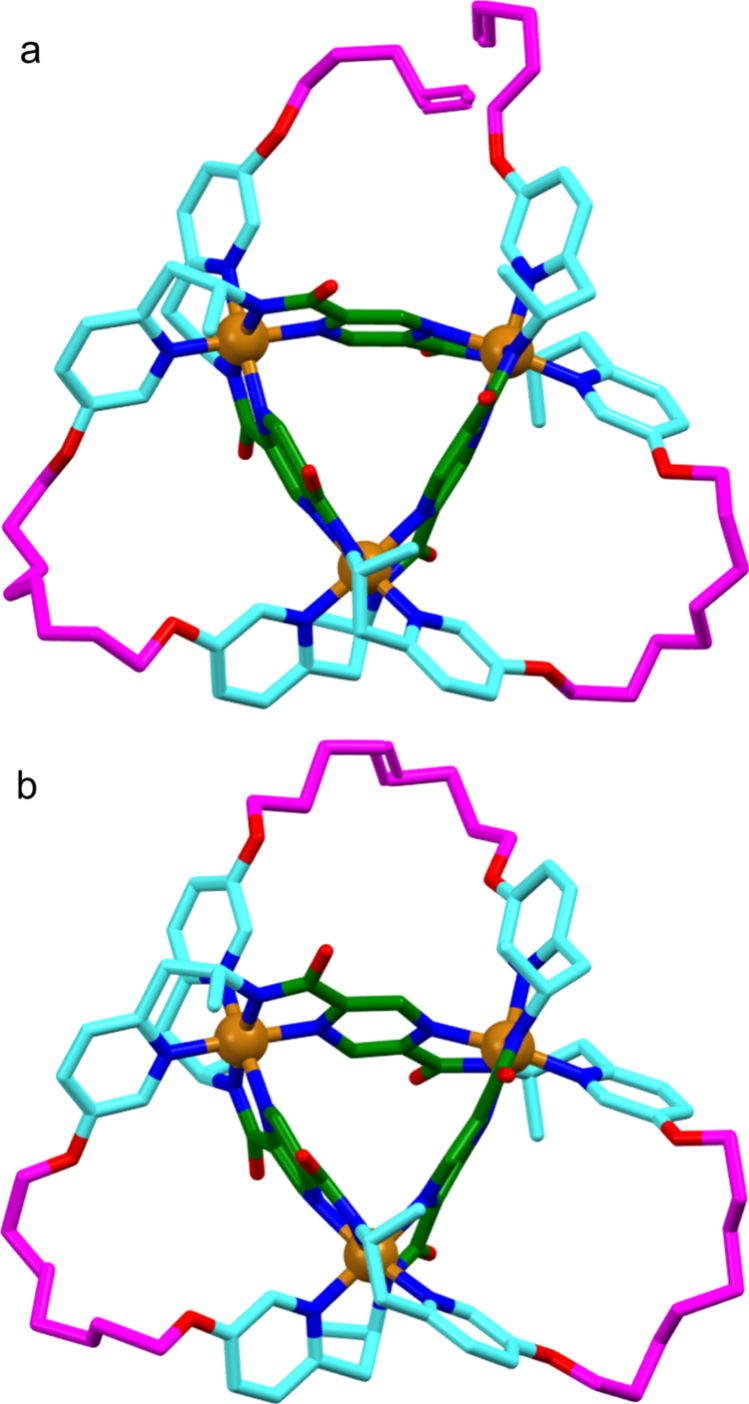
X-ray crystal structures of (a) overhand knot complex Δ-**2**·[Co_3_] and (b) trefoil knot complex Δ-**3**·[Co_3_]. Hydrogen atoms, solvent molecules, and anions are omitted for clarity. Atom colors: nitrogen, blue; cobalt, orange; oxygen, red; carbons from the alkyl chain linkers, magenta; carbons from the pyrazine-2,5-dicarboxamides, green; carbons from the 2-pyridylethylamine units, turquoise.

Single crystals of Δ-**2**·[Co_3_] were obtained by slow diffusion of diethyl ether into an acetonitrile solution of the complex and the solid-state structure determined by X-ray diffraction (Figure 3a). The trinuclear compound crystallizes in the chiral *P*321 space group. The crystal structure (Figure 3a and Supporting Information) confirms the formation of an overhand knot, with the absolute configuration Δ (Flack parameter = zero). The organic ligand strand wraps around three cobalt centers and passes over or under itself at each cobalt atom. All of the cobalt cations are located in a distorted octahedral geometry, coordinating with six nitrogen atoms: two pyrazines, two pyridines, and two amides. The metal–ligand bond lengths are shorter than those of a circular helicate^14^ obtained from the self-assembly of three monomeric ligands (N_pz_–Co [average] 1.98(3) *vs* 2.02(2); N_py_–Co [average] 1.94(3) *vs* 2.01(4); N_amide_–Co [average] 1.93(3) *vs* 1.97(4)) in order to adopt a geometry closer to an equilateral triangle.

To connect the ligand termini of the overhand knot, Δ-**2**·[Co_3_] was treated with the Hoveyda-Grubbs second-generation catalyst^15^ in a 1:3 mixture of nitromethane and 1,2-dichloroethane at 60 °C for 48 h. The reaction was quenched with ethyl vinyl ether, followed by precipitation with diethyl ether, to afford the corresponding trefoil knot complex Δ-**3**·[Co_3_] in near-quantitative yield (Scheme 2, step b). The mass spectrum of Δ-**3**·[Co_3_] confirmed the loss of one ethene molecule from the overhand knot (*m*/*z* Δ-**3**·[Co(III)_3_]^3+^ 602.83, Δ-**3**·[Co(III)_3_][BF_4_]^2+^ 947.75 etc.; Supporting Information Figure S3), with excellent correlation between the observed and the calculated isotopic distributions (Supporting Information Figure S4).

An additional consequence of the ring closing of the knot is that the majority of cobalt ions in the trefoil knot complex appear to have been oxidized to Co(III), presumably by air during the recrystallization process. This may be due to strain in the knot geometry, destabilizing the lower metal oxidation state. The ^1^H NMR spectrum of Δ-**3**·[Co_3_] in CD_3_CN (Figure 2c) is much sharper than that of the overhand knot Δ-**2**·[Co_3_] (Figure 2b). The olefin region of the ^1^H NMR spectrum of Δ-**3**·[Co_3_] shows that the terminal alkene signals (H_n_ and H_o_; Figure 2b) of the overhand knot complex have been replaced by those of an internal alkene (Figure 2c). The more pronounced separation of the diastereotopic H_g_ and H_k_ protons may be a result of the increased conformational restriction of the chains in the closed-loop knot Δ-**3**·[Co_3_] compared to that in Δ-**2**·[Co_3_]. DOSY spectroscopy confirmed that Δ-**3**·[Co_3_] is a single discrete species (Supporting Information Figure S27).

Slow diffusion of diethyl ether into a solution of Δ-**3**·[Co_3_] in acetonitrile afforded single crystals suitable for X-ray diffraction. The solid-state structure (Figure 3b and Supporting Information) confirms the topology of the molecular trefoil knot and confirms it to be one topological enantiomer (Δ-configuration). The 90-atom-long closed loop weaves a continuous path passing under and over itself 3 times to form a *pseudo-*D_3_-symmetric trefoil knot. Since the ^1^H NMR data shows both *E*- and *Z*-olefins present in the sample used to grow the single crystal used for crystallography, both stereoisomers were modeled (1:1 ratio) in the highly disordered linker region of the chains.

Trefoil knot complex Δ-**3**·[Co_3_] was demetalated by treatment of a solution of Δ-**3**·[Co_3_] methanol:acetic acid (1:1) with activated zinc dust, followed by washing with a 17.5% NH_3_ solution saturated with tetrasodium ethylenediaminetetraacetate (Na_4_EDTA). The loss of the dark brown color from the solution indicated the decoordination of the Co(III) ions. After workup, the reaction mixture was subjected to hydrogenation with H_2_ over Pd/C to reduce the double bond,^16^ giving the wholly organic trefoil knot Δ-**4** in 35% yield after purification by preparative TLC (Scheme 2, step b). The composition of the metal-free knot was confirmed by matrix-assisted laser desorption/ionization–time-of-flight (MALDI-TOF) mass spectrometry (Supporting Information Figures S5 and S6), HRESI-MS (Supporting Information Figure S7), and ^1^H NMR spectroscopy (Figure 2d). Substantial upfield shifts of the resonances for H_d_ and H_f_ are apparent in the ^1^H NMR spectrum of the knot (Figure 2d) compared with ligand strand **1** (Figure 2a), consistent with CH-π interactions between different regions of the constrained knotted loop. Note that the signals in the ^1^H NMR of the demetalated knot are well resolved (Figure 2d). This is because, without coordination to metal centers, there is rapid reptation^17^ of the knotted backbone leading to a time-averaged signal of all of the sampled knot conformations.^7h,7k,10h−10j^

**Figure 4:**
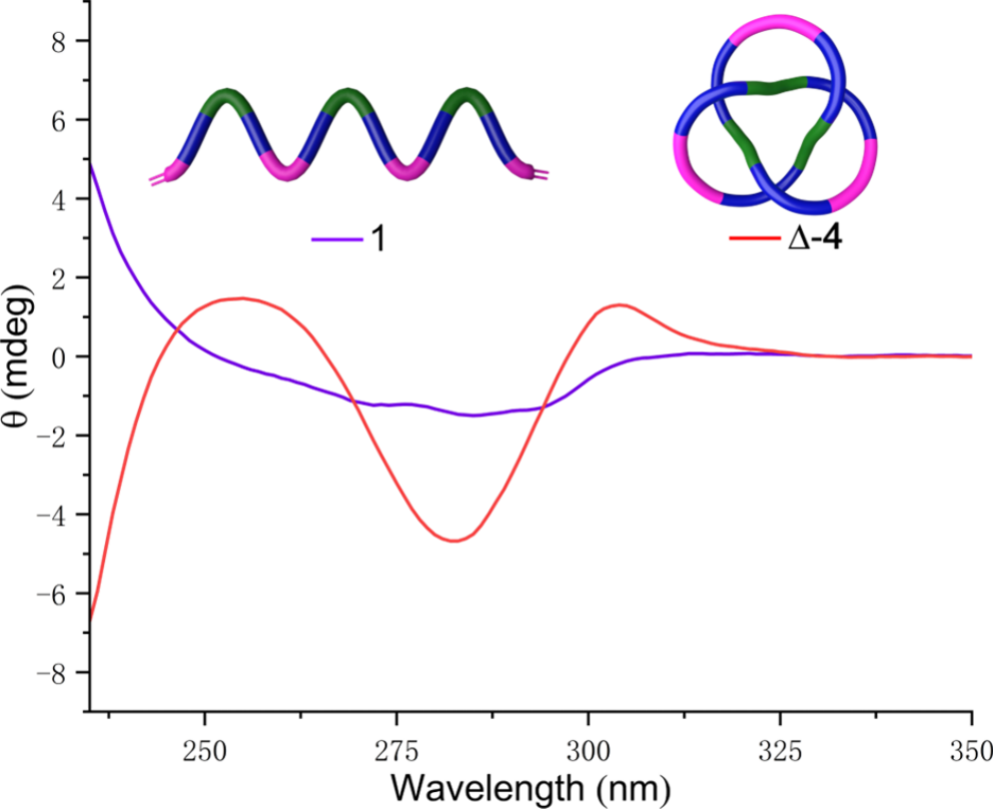
CD spectra (0.05 mM, CH_2_Cl_2_, 298 K) of ligand strand **1** (magenta) and metal-free trefoil knot Δ-**4** (red). Normalized for absorbance.

The metal-free knot was also characterized by circular dichroism (Figure 4). The ultraviolet–visible (UV–vis) absorption spectra of ligand **1** and knot Δ-**4** have similar profiles with an absorption band at 283 nm (Supporting Information Figure S8) in the expected range for pyrazine-2,5-dicarboxamides.^14^ However, the CD spectrum of the trefoil knot Δ-**4** shows much more pronounced absorption in the range 254–334 nm than that of chiral ligand **1** (Figure 4). In Δ-**4**, topological chirality has a much greater impact on the asymmetry of the chromophore environment than the point-chirality of the asymmetric carbon atoms in the covalent framework of the knot. The shape of the CD spectrum of the knot is consistent with Δ-handed topological chirality.^13^

## Conclusions

A single molecular strand can be efficiently folded into an overhand knot of single handedness through the formation of a circular helicate with three coordinatively dynamic Co(II) ions that are then kinetically locked in place by oxidation to Co(III). Oxidation of all of the metals is unnecessary to maintain the structural integrity of the folded helicate, as even one Co(III) ion binding to the correct three chelating groups of the strand would prevent the circular helicate from unraveling or crossings being undone. Ring-closing olefin metathesis can be used to connect the end groups of the strand, forming a closed-loop trefoil knot. Point-chiral carbon centers on the strand ensure that the formed molecular knot is of single topological handedness. The effectiveness of the “chaperone”-driven folding process, and the kinetic stability of the intermediate Co(III) overhand knot, opens the way for the synthesis of more complex knot topologies and the exploration of the effects^7^ of entanglements (both ordered and randomly formed) on molecular and polymer properties.

## Supporting Information Available

Experimental details and methods; characterization data including electronic absorption; circular dichroism; NMR; MS; and X-ray crystallography data (PDF)
