# Supplementary material for: Folding a Molecular Strand into a Trefoil Knot of Single Handedness with Co(II)/Co(III) Chaperones
Source: J Am Chem Soc. 2024 Jul 26;146(31):21762–8. doi: 10.1021/jacs.4c05953 (PMC11311214; doi:10.1021/jacs.4c05953)
Supplement: Supplementary file 1 — ja4c05953_si_001.pdf [file ja4c05953_si_001.pdf]

Supporting Information

Folding a Molecular Strand into a Trefoil Knot of Single Handedness with Co(II)/Co(III) Chaperones

Jiankang Zhong[†], Zhanhu Sun[‡], Liang Zhang[‡], George F. S. Whitehead,[‡] Iñigo J. Vitorica-Yrezabal[‡] and David A. Leigh[†,‡,*]

[‡]School of Chemistry and Molecular Engineering, East China Normal University, 200062 Shanghai, China.

[†]Department of Chemistry, University of Manchester, Manchester M13 9PL, UK.

Email: david.leigh@manchester.ac.uk

Table of Contents

1. General Experimental Section S3
2. Synthetic Overview S4
3. Synthetic Procedures and Characterization Details S6
 3.1 Synthesis of ligand **1** S6
 3.2 Synthesis of homochiral overhand knot Δ-**2**·[Co_3] S10
 3.3 Synthesis of homochiral trefoil knot Δ-**3**·[Co_3] S12
 3.4 Synthesis of wholly organic trefoil knot Δ-**4**. S14
4. CD and UV/Vis Spectra S17
5. X-ray Crystal Structure Information S18
6. NMR Spectra S22
7. Molecular Modelling (DFT level) of Trefoil Knot Δ-**4** S31
8. References S33

1. General Experimental Section

All reagents and solvents were purchased from Sigma-Aldrich Chemicals and used without further purification. Compound **5** and ligand **1** were prepared according to literature procedures.[S1,S2,S3] Where stated, procedures were carried out using dry solvents and these were obtained by passing through an activated alumina column on a Phoenix SDS solvent drying system (JC Meyer Solvent Systems, CA, USA). Unless stated otherwise, all procedures were carried out under an inert atmosphere of N_2. NMR spectra were recorded on a BrukerAvance III (equipped with a cryoprobe) instrument with an Oxford AS600 magnet. Chemical shifts are reported in parts per million (ppm) from high to low frequency and referenced to the residual solvent resonance. N^{th} order coupling constants (^{n}J) are reported in Hertz (Hz). Standard abbreviations indicating multiplicity were used as follows: s = singlet, d = doublet, t = triplet, q = quartet, quin = quintet, m = multiplet, br = broad. ^{1}H assignments were made using 2D NMR methods (COSY, HSQC, HMBC). Low resolution ESI mass spectrometry was performed with a Thermo Scientific LCQ Fleet or an Agilent Technologies 1200 LC system with 6130 single quadrupole MS detector mass spectrometer. High resolution ESI (electrospray ionization) and MALDI (matrix assisted LASER desorption ionization) mass spectrometry were carried out by the mass spectrometry services at the University of Manchester. All circular dichroism (CD) spectra were recorded on a J-815 Jasco spectrometer (Jasco France, Nantes, France). Spectra were acquired in spectrophotometric grade solvents using a quartz cell with a path length of 1 mm.

2. Synthetic Overview

Scheme S1. Synthesis of target ligand **1**

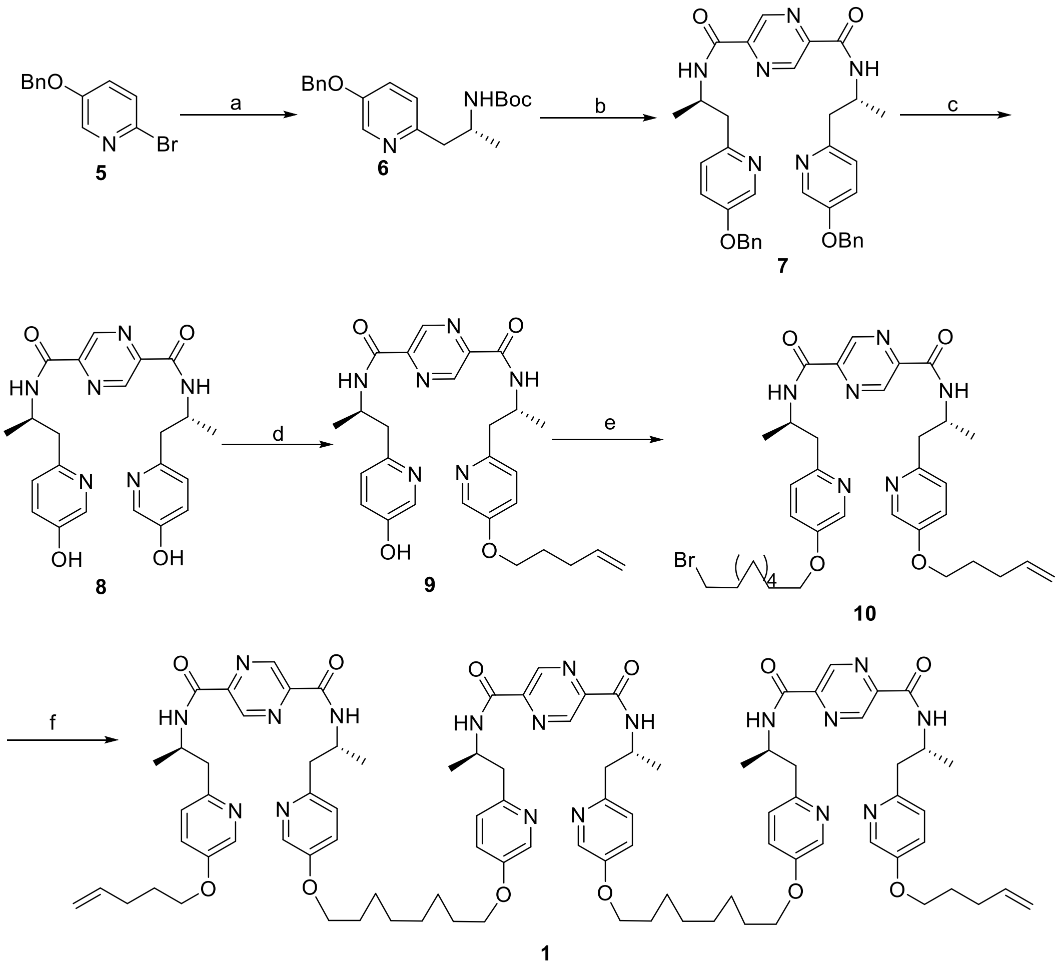

[a] Reagents and conditions: a) Zn, 1,2-dibromoethane, TMSCl, (*R*)-*tert*-butyl(1-iodopropan-2-yl)carbamate, $Pd(PPh_3)_2Cl_2$, DMF, 0 °C to 45 °C, 48 h, 75%; b) TFA, DCM, 0 °C to rt, 12 h; then pyrazine-2,5-dicarbonyl chloride, Et_3N, DCM, 0 °C to rt , overnight, 85%; c) H_2, Pd/C, MeOH, 45 °C, 2 h, quant.; d) 5-bromo-1-pentene, K_2CO_3, DMF, 60 °C for 1 h, then r.t., 3 d, 47%; e) 1,8-dibromo-octane, K_2CO_3, DMF, 50 °C, 18 h, 87%; f) **8**, K_2CO_3, DMF, 50 °C, 3 d, 65%.

Scheme S2. Synthesis of an enantiopure Co molecular trefoil knot **3** and the corresponding organic trefoil knot **4**[a]

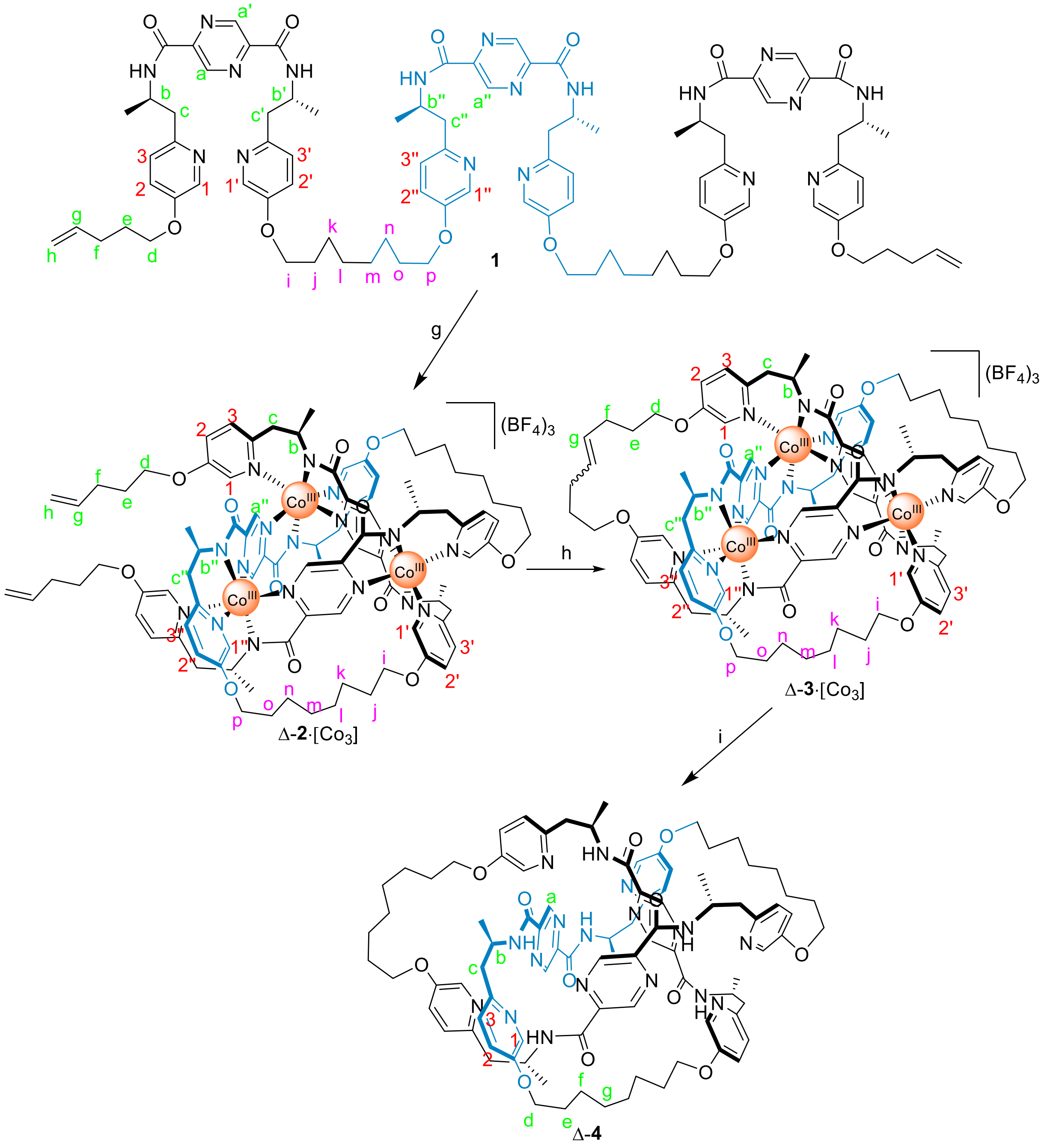

[a]Reagents and conditions: (g) $Co(BF_4)_2{\cdot}6H_2O$, Et_3N, acetonitrile, rt to 85 °C, 36 h, then O_2, 80 °C, 3 days, 85 %; (h) Hoveyda-Grubbs second generation catalyst, 1,2-dichloroethane/nitromethane (3:1), 60 °C, 48 h, >98%; (i) Zinc metal, CH_3COOH/MeOH (1/1), rt, 2 h, then H_2, Pd/C, CH_2Cl_2/MeOH (1/1), 40 °C, 24 h, 35 %.

3. Synthetic Procedures and Characterization Details

3.1 Synthesis of ligand **1**

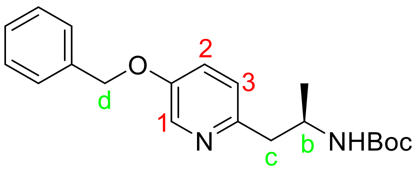

6

Zinc dust (1.59 g, 24.3 mmol) was added to a two-necked flask, purged with N_2 and heated with a heat gun for 10 min under vacuum. The slurry was cooled to rt and a solution of 1,2-dibromoethane (105 uL, 1.22 mmol) in dry DMF was added dropwise. The resulting suspension was heated to 90 °C for 30 min and cooled to rt. TMSCl (35 uL, 0.27 mmol) was added and the suspension was stirred for a further 30 min at rt. A solution of (*S*)-tert-butyl (1-iodopropan-2-yl)carbamate (1.5 g, 5.26 mmol) in dry DMF was added dropwise at 0 °C and stirred for 90 min at 0 °C. Then **5** (1.2 g, 5.26 mol) and $PdCl_2(PPh_3)_2$ (153 mg, 0.22 mmol) were added and continued to stir at 0 °C overnight. The mixture was diluted with EtOAc and filtered through celite, and the combined organic layers were concentrated to dryness. The residue was purified by flash chromatography, eluting with Hexane/EtOAc (10:1/1:1) to give **6** (1.35 g, 75%) as a pale yellow solid. ^1H NMR (600 MHz, $CDCl_3$) δ 8.30 (d, *J* = 2.9 Hz, 1H, H_1), 7.45 – 7.31 (m, 5H, Ph), 7.22 (dd, *J* = 8.5, 3.0 Hz, 1H, H_2), 7.11 (d, *J* = 8.4 Hz, 1H, H_3), 5.10 (br, 1H, NH), 5.09 (s, 2H, H_d), 3.99 (m, 1H, H_b), 2.96 – 2.84 (m, 2H, H_c), 1.40 (s, 9H, Boc), 1.13 (d, *J* = 6.6 Hz, 3H, Me). ^{13}C NMR (151 MHz, $CDCl_3$) δ 155.42, 153.65, 151.16, 136.28, 132.28, 128.87, 128.46, 127.68, 124.24, 70.62, 47.03, 43.55, 28.55, 20.72. HRESI-MS: *m/z* = 342.1945 $[M+H]^+$, (calcd. for $C_{20}H_{26}N_2O_3$, 342.1943).

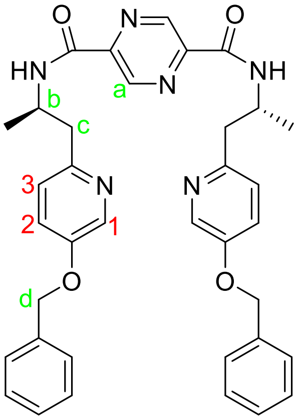

7

To a stirred solution of **6** (283 mg, 0.68 mmol) in dry DCM (6.8 mL) at 0 °C, TFA (1.36 mL) was added. The resulting mixture was stirred at room temperature for 2 h. After this time, the resulting mixture was concentrated to dryness, then diluted with EtOAc and partitioned with

saturated $NaHCO_3$ solution (5 mL). The aqueous phase was extracted with EtOAc (4 x 5 mL) and the combined organic phase was dried (Na_2SO_4), filtered and concentrated under reduced pressure to give a yellow oil. The yellow oil was taken up in dry DCM (4 mL), and Et_3N (0.38 mL, 2.72 mmol) was added to this solution, followed by slow addition of a solution of pyrazine-2,5-dicarbonyl dichloride (70 mg, 0.34 mmol) in dry DCM (2.8 mL) under N_2 at 0 °C. The reaction mixture was stirred at room temperature overnight, then concentrated to dryness. The residue was purified by flash chromatography, eluting with DCM/MeOH (40:1/20:1) to give **7** (179 mg, 85%) as a colourless solid. ^{1}H NMR (600 MHz, $CDCl_3$) δ 9.28 (s, 2H, H_a), 8.56 (d, J = 8.2 Hz, 2H, NH), 8.33 (d, J = 2.9 Hz, 2H, H_1), 7.44 – 7.35 (m, 8H, Ph), 7.35 – 7.30 (m, 2H, Ph), 7.19 (dd, J = 8.5, 3.0 Hz, 2H, H_2), 7.12 (d, J = 8.5 Hz, 1H, H_3), 5.08 (s, 4H, H_d), 4.60 – 4.52 (m, 2H, H_b), 3.06 (dd, J = 13.9, 5.7 Hz, 2H, H_c), 2.99 (dd, J = 13.9, 6.5 Hz, 2H, $H_{c'}$), 1.29 (d, J = 6.6 Hz, 6H, Me). ^{13}C NMR (151 MHz, $CDCl_3$) δ 161.88, 153.69, 150.67, 146.52, 142.29, 137.52, 136.29, 128.84, 128.42, 127.67, 124.19, 122.51, 116.53, 70.53, 46.11, 42.91, 20.26. HRESI-MS: m/z = 617.2877 $[M+H]^+$, (calcd. for $C_{36}H_{37}N_6O_4$, 617.2871).

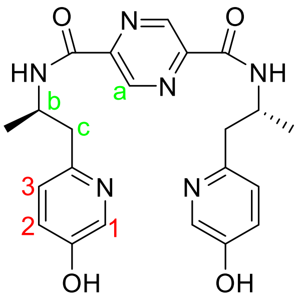

8

A solution of **7** (305 mg, 0.5 mmol) in 20 mL MeOH was treated with 5% Pd/C (152 mg), and the resulting black suspension was heated at 45 °C under H_2 atmosphere using a H_2 bubble for 2 h. The catalyst was removed by filtration through a pad of celite, and the filtrate was evaporated to provide the target product **8** in quantitative yield. ^{1}H NMR (600 MHz, CD_3OD) δ 9.17 (s, 2H, H_a), 8.00 (d, J = 2.7 Hz, 2H, H_1), 7.18 (d, J = 8.4 Hz, 2H, H_3), 7.12 (dd, J = 8.4, 2.8 Hz, 2H, H_2), 4.53 – 4.46 (m, 2H, H_b), 3.01 – 2.94 (m, 4H, H_c), 1.28 (d, J = 6.7 Hz, 6H, Me). ^{13}C NMR (151 MHz, CD_3OD) δ 163.85, 154.28, 150.10, 147.93, 143.27, 137.67, 125.91, 124.84, 47.76, 43.69, 20.38. HRESI-MS: m/z = 437.1935 $[M+H]^+$, (calcd. for $C_{22}H_{25}N_6O_4$, 437.1932).

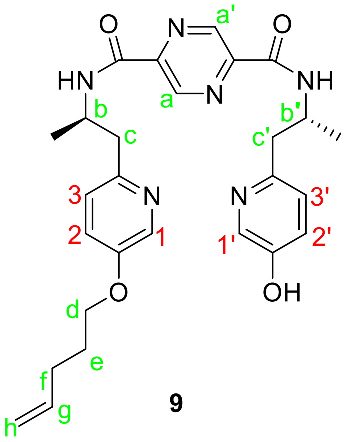

9

Diol **8** (186 mg, 0.426 mmol) was taken up in degassed DMF (20 mL) and K_2CO_3 (59 mg, 0.426 mmol) was added. The reaction mixture was stirred at 60 °C for 1 h and cooled to room temperature. Subsequently, a solution of 5-bromo-1-pentene (51 µL, 0.426 mmol) in degassed DMF (10 mL) was slowly added into the reaction solution over 2 h and the reaction solution was stirred at room temperature for 2 days. The resulting mixture was concentrated to dryness. The residue was purified by flash chromatography, eluting with ethyl acetate to provide the target product **9** (100 mg, 47%) as a colourless solid. ^{1}H NMR (600 MHz, $CDCl_3$) δ 9.24 (m, 2H, $H_{a,a'}$), 8.56 (d, *J* = 8.2 Hz, 1H, NH), 8.51 (d, *J* = 8.2 Hz, 1H, NH), 8.22 (d, *J* = 2.3 Hz, 2H, $H_{1'}$), 8.15 (d, *J* = 2.3 Hz, 2H, H_1), 7.15 – 7.07 (m, 4H, $H_{2,2',3,3'}$), 5.83 (ddt, *J* = 17.0, 10.1, 6.7 Hz, 1H, H_g), 5.13 – 4.96 (m, 2H, H_h), 4.59 – 4.49 (m, 2H, $H_{b,b'}$), 3.99 (t, *J* = 6.4 Hz, 2H, H_d), 3.10 – 2.94 (m, 4H, $H_{c,c'}$), 2.26 – 2.21 (m, 2H, H_f), 1.91 – 1.85 (m, 2H, H_e), 1.30 (d, *J* = 6.6 Hz, 3H, Me), 1.28 (d, *J* = 6.6 Hz, 3H, Me). ^{13}C NMR (151 MHz, $CDCl_3$) δ 161.97, 161.94, 154.11, 151.74, 150.07, 149.86, 146.47, 146.45, 142.25, 137.66, 137.33, 137.00, 124.51, 124.40, 123.86, 122.25, 115.60, 67.75, 46.28, 46.14, 42.85, 42.75, 30.11, 28.43, 20.42, 20.22. HRESI-MS: *m/z* = 527.2372 $[M+Na]^+$, (calcd. for $C_{27}H_{32}N_6O_4$, 527.2377).

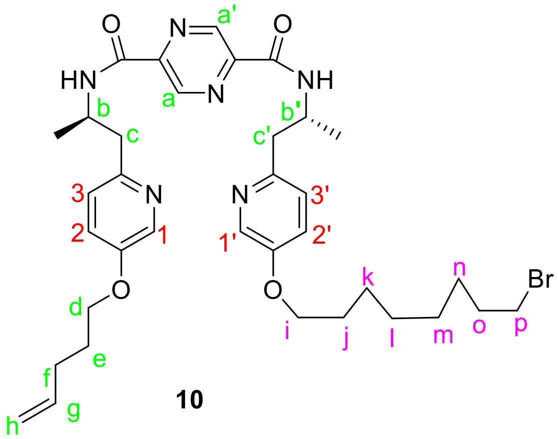

10

To a stirred solution of **9** (100 mg, 0.198 mmol) in degassed DMF (6 mL) in the presence of K_2CO_3 (82 mg, 0.594 mmol) was added 1,8-dibromo-octane (161 µL, 0.792 mmol) under N_2. The reaction mixture was stirred at 50 °C for 18 h. Then the reaction mixture was diluted with DCM and washed with 5 % LiCl solution (3 x 5 mL), then the aqueous phase was extracted

with DCM (2 x 5 mL). The combined organic phases were dried over Na_2SO_4 and concentrated to dryness. The residue was purified by flash chromatography, eluting with ethyl acetate to provide target product **10** (120 mg, 87%) as a colourless solid. ^{1}H NMR (600 MHz, $CDCl_3$) δ 9.29 (s, 2H, $H_{a,a'}$), 8.63 – 8.58 (m, 2H, NH), 8.26 – 8.23 (m, 2H, $H_{1,1'}$), 7.13 – 7.10 (m, 4H, $H_{2,2',3,3'}$), 5.84 (ddt, *J* = 16.9, 10.2, 6.7 Hz, 1H, H_g), 5.12 – 4.96 (m, 2H, H_h), 4.58 – 4.51 (m, 2H, $H_{b,b'}$), 4.00 – 3.95 (m, 2H, $H_{d,i}$), 3.41 (t, *J* = 6.8 Hz, 2H, H_p), 3.09 – 2.96 (m, 4H, $H_{c,c'}$), 2.26 – 2.21 (m, 2H, H_f), 1.92 – 1.83 (m, 4H, $H_{e,j}$), 1.81 – 1.75 (m, 2H, H_o), 1.49 – 1.42 (m, 4H, $H_{k,n}$), 1.39 – 1.34 (m, 4H, $H_{l,m}$), 1.28 (d, *J* = 6.6 Hz, 6H, Me). ^{13}C NMR (151 MHz, $CDCl_3$) δ 161.80, 153.95, 153.90, 150.14, 150.07, 146.46, 137.59,137.16, 132.11, 124.09, 121.93, 115.49, 68.38, 67.62, 46.04, 42.73, 34.06, 32.81, 30.03, 29.76, 29.20, 28.72, 28.36, 28.12, 25.92, 20.14; due to high degree of apparent symmetry, several ^{13}C signals overlap. HRESI-MS: *m/z* = 717.2724 $[M+Na]^+$, (calcd. for $C_{35}H_{47}O_4N_6BrNa$, 717.2734).

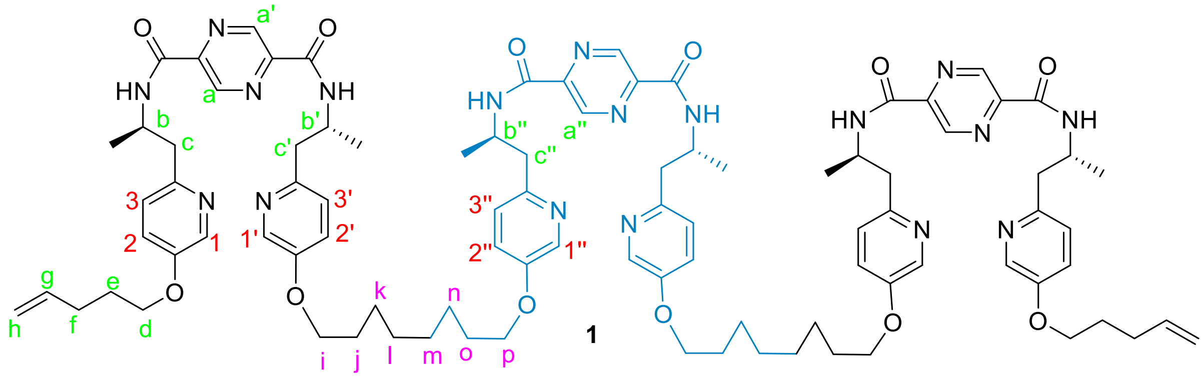

1

Bromide **10** (108 mg, 0.154 mmol) and diol **8** (30 mg, 0.07 mmol) were dissolved in degassed DMF (14 mL). K_2CO_3 (48 mg, 0.35mmol) was added and the reaction mixture was heated to 50 °C and stirred under nitrogen for 3 days. Upon completion, the reaction mixture was diluted with DCM and washed with 5 % LiCl solution (3 x 5 mL), then the aqueous phase was extracted with DCM (3 x 5 mL). The combinded organic phases were dried over Na_2SO_4 and concentrated to dryness. The residue was purified by flash column chromatography, eluting with EA then CH_2Cl_2/MeOH (15:1 to 10:1) to afford chiral ligand **1** (76 mg, 65%) as a colourless solid. ^{1}H NMR (600 MHz, $CDCl_3$) δ 9.31 – 9.26 (m, 6H, $H_{a,a',a''}$), 8.64 – 8.58 (m, 6H, NH), 8.26 – 8.22 (m, 6H, $H_{1,1',1''}$), 7.15 – 7.07 (m, 12H, $H_{2,2',2'',3,3',3''}$), 5.86 – 5.79 (m, 2H, H_g), 5.08 – 5.03 (m, 2H, H_h), 5.02 – 4.97 (m, 2H, $H_{h'}$), 4.58 – 4.50 (m, 6H, $H_{b,b',b''}$), 4.03 – 3.91 (m, 12H, $H_{d,i,p}$), 3.10 – 2.94 (m, 12H, $H_{c,c',c''}$), 2.26 – 2.20 (m, 4H, H_f), 1.91 – 1.85 (m, 4H, H_e), 1.82 – 1.74 (m, 8H, $H_{j,o}$), 1.50 – 1.43 (m, 8H, $H_{k,n}$), 1.41 – 1.36 (m, 8H, $H_{l,m}$), 1.29 – 1.24 (m, 18H, Me). ^{13}C NMR (151 MHz, $CDCl_3$) δ 161.87, 154.02, 153.96, 150.22, 150.13, 146.53, 142.28, 137.65, 137.23, 137.18, 124.17, 124.15, 121.98, 115.55, 68.48, 67.68, 46.09, 42.79, 42.75, 30.10, 29.41, 29.30, 29.30, 28.43, 26.05, 20.19, 20.18, 20.13; due to high degree of apparent symmetry, several ^{13}C signals overlap. HRESI-MS: *m/z* = 1665.9065 $[M+H]^+$, (calcd. for $C_{92}H_{117}N_{18}O_{12}$, 1665.9093).

3.2 Synthesis of homochiral overhand trefoil knot Δ-**2**

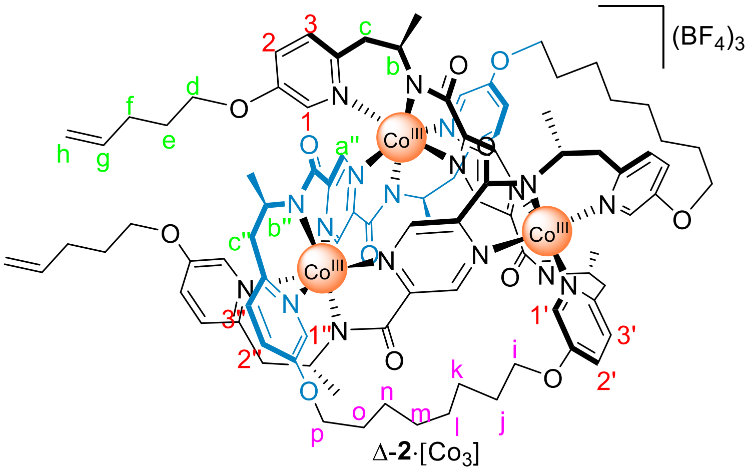

Δ-**2**·[Co3]

To a stirred solution of **1** (27 mg, 0.0162 mmol) in degassed acetonitrile (15 mL) at room temperature was added a degassed CH_3CN (0.2 mL) solution of cobalt (II) tetrafluoroborate hydrate (20.4 mg, 0.0583 mmol) and Et_3N (20 μL, 0.146 mmol). The reaction mixture was stirred at 85 °C for 36 h. After that O_2 was bubbled through the solution at 80 °C for 3 days. After cooling to room temperature, the reaction mixture was concentrated to dryness, the residue was dissolved in a small amount of CH_3CN and transferred to a centrifugal tube and a small amount of diethyl ether added. The tubes were centrifuged (ca. 55 rpm, 5 min). The supernatant was decanted off, and the precipitate was concentrated to dryness under vacuum to give the crude compound Δ-**2**·[Co_3], which was recrystallized via slow diffusion diethyl ether into saturated CH_3CN solution of the crude mixture to give the pure compound Δ-**2**·[Co_3] (28 mg, 85%). ^{1}H NMR (600 MHz, CD_3CN) δ 8.70 – 8.39 (br, 6H, $H_{a,a',a''}$), 7.69 – 7.39 (br, 18H, $H_{2,2',2'',3,3',3'',1,1',1''}$), 5.86 – 5.70 (br, 2H, H_g), 5.03 – 4.63 (br, 10H, $H_{h,b,e',e''}$), 4.16 – 3.66 (br, 12H, $H_{d,i,p}$), 3.20 – 2.76 (br, 12H, $H_{c,c',c''}$), 2.20 – 1.99 (br, 8H, $H_{e,f}$), 1.79 – 1.57 (br, 8H, $H_{j,o}$), 1.50 – 1.00 (br, 34H, $H_{k,l,m,n,Me}$). ^{13}C NMR (151 MHz, CD_3CN) δ 168.70, 168.67, 168.64, 156.03, 155.96, 155.82, 154.93, 154.91, 154.89, 154.03, 153.77, 153.58, 148.07, 148.00, 147.88, 144.63, 144.62, 143.35, 143.33, 138.47, 130.99, 130.91, 130.83, 127.93, 126.24, 115.70, 113.99, 69.91, 69.54, 69.50, 48.10, 48.01, 47.91, 42.14, 42.05, 30.34, 30.13, 29.92, 28.63, 28.46, 28.30, 20.00, 19.99; due to high degree of apparent symmetry, several ^{13}C signals overlap. HRESI-MS: m/z = 917.8286 [$Co^{III}_2Co^{II}$ -2BF_4]$^{2+}$ requires 917.8268.

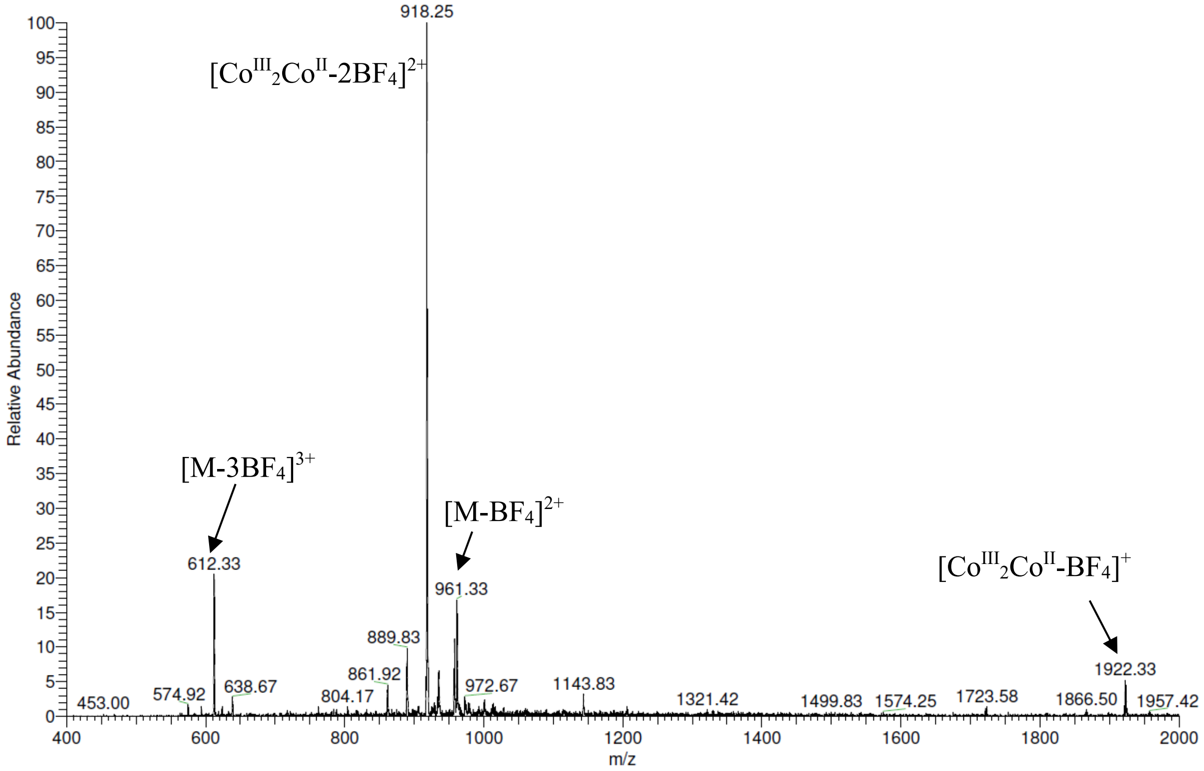

Figure S1: Low-resolution ESI-MS of Co overhand knot Δ-**2**·[Co_3]. Calculated peaks (*m/z*): 1922.6570 [$Co^{III}{}_2Co^{II}$-BF_4]$^+$; 961.3288 [M-2BF_4]$^{2+}$; 917.8268 [$Co^{III}{}_2Co^{II}$-2BF_4]$^{2+}$; 611.8843 [M-3BF_4]$^{3+}$. Partial reduction of the compounds was apparent under these ESI-MS conditions.

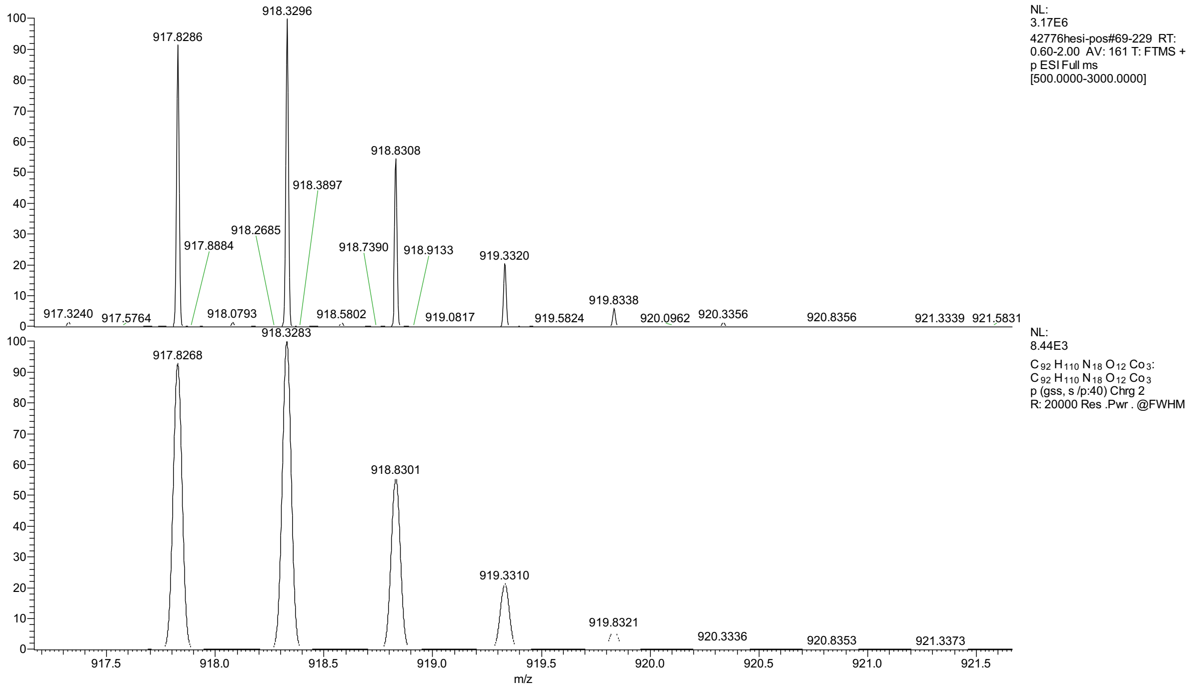

Figure S2: High-resolution ESI-MS of the [$Co^{III}Co^{II}{}_2$-2BF_4]$^{2+}$ *m/z* ion. Experimental spectrum (top) and calculated spectrum (bottom).

3.2 Synthesis of homochiral trefoil knot Δ-**3**·[Co_3]

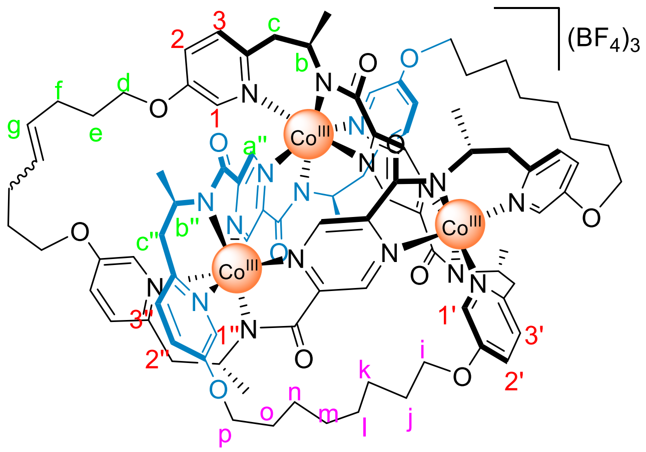

Δ-**3**·[Co_3]

A solution of Δ-**2**·[Co_3] (34.0 mg, 16.2 μmol) in a mixture of degassed anhydrous nitromethane (4.0 mL) and degassed anhydrous1,2-dichloroethane (8.0 mL) was added to an oven dried flask equipped with a stirrer bar under N_2. Hoveyda-Grubbs 2nd generation catalyst (H_2IMes)(Cl)$_2$RuCH(o-O*i*PrC_6H_4) (10.2 mg, 16.2 μmol, 100 mol%) in degassed anhydrous 1,2-dichloroethane (4.0 mL) was added, and the reaction heated to 60 °C for 48 hours with stirring. After cooling to room temperature, the catalyst was quenched with excess ethylvinyl ether (0.4 mL) and stirred for 30 minutes. The solvent was removed under reduced pressure and the residue was dissolved in a small amount of CH_3CN and transferred to a centrifugal tube and a small amount of diethyl ether was added. The tubes were centrifuged (ca. 55 rpm, 5 min). The supernatant was decanted off, and the precipitate was concentrated to dryness under vacuum to give the crude compound Δ-**3**·[Co_3], which was recrystallized via slow diffusion of diethyl ether into saturated CH_3CN solution of the crude mixture to give the pure compound Δ-**3**·[Co_3] (34 mg, Quant.). ^{1}H NMR (600 MHz, CD_3CN) δ 8.59 – 8.56 (m, 6H, $H_{a,a',a''}$), 7.56 – 7.44 (m, 8H, $H_{2,2',2'',3}$), 7.43 – 7.35 (m, 4H, $H_{3',3''}$), 7.32 – 7.24 (m, 6H, $H_{1,1',1''}$), 5.40 – 5.37 (m, 1H, $H_{g'}$), 5.35 (t, J = 4.8 Hz, 1H, H_g), 4.93 – 4.71 (m, 6H, $H_{b,b',b''}$), 4.11 – 3.99 (m, 4H, $H_{i,k}$), 3.96 – 3.74 (m, 8H, $H_{i',k',d}$), 3.06 – 2.87 (m, 6H, $H_{c,c',c''}$), 2.16 – 1.96 (m, 8H, $H_{e,f}$), 1.73 – 1.54 (m, 8H, $H_{j,o}$), 1.41 – 1.11 (m, 34H, $H_{k,l,m,n,Me}$). ^{13}C NMR (151 MHz, CD_3CN) δ 168.80, 168.74, 156.32, 156.02, 155.98, 154.08, 154.04, 153.90, 153.81, 153.62, 153.39, 148.13, 148.10, 147.86, 144.68, 144.64, 143.57, 130.87, 130.83, 130.79, 130.75, 130.70, 126.28, 126.25, 126.10, 69.57, 69.53, 69.28, 48.18, 48.02, 47.93, 42.17, 42.09, 41.98, 28.66, 28.64, 28.58, 28.56, 28.37, 28.23, 28.16, 27.53, 26.88, 26.86, 26.54, 26.53, 26.51, 25.97, 23.29, 21.98, 21.58, 20.05, 20.02, 19.98, 19.94, 19.90; due to high degree of apparent symmetry, several ^{13}C signals overlap. HRESI-MS: m/z = 903.8130 [$Co^{III}_2Co^{II}$-2BF_4]$^{2+}$ requires 903.8111.

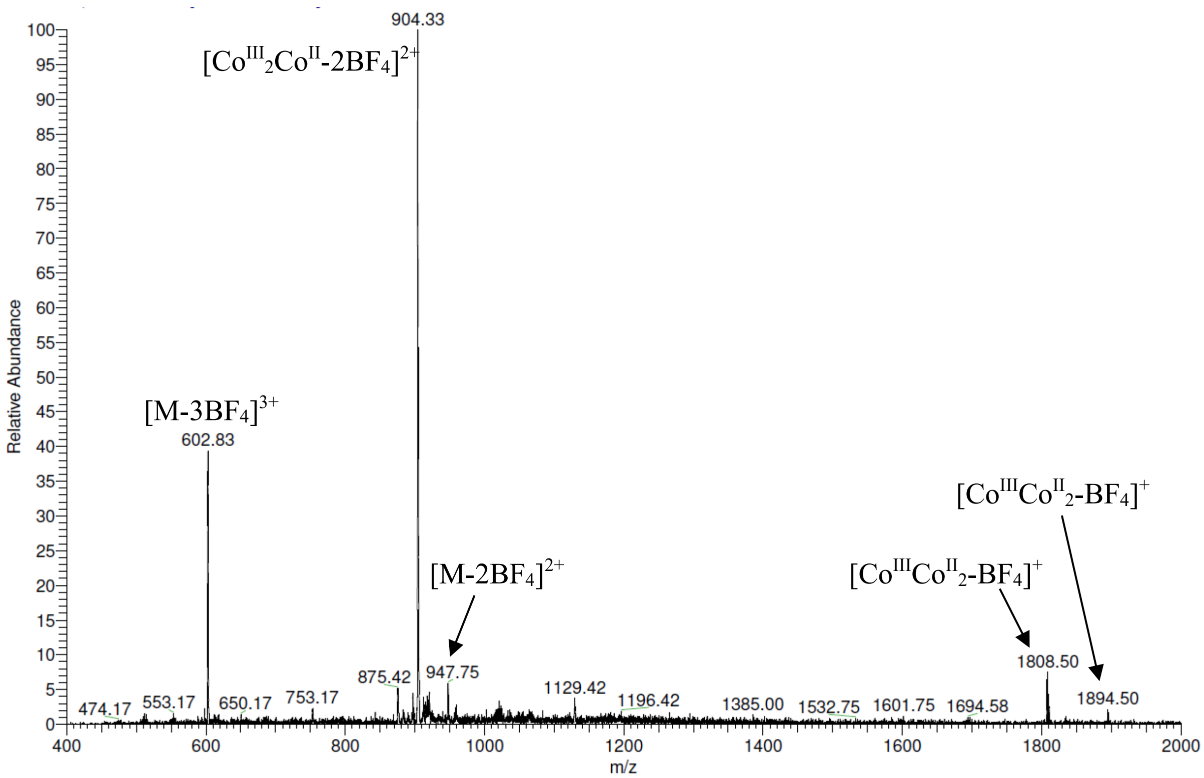

Figure S3: Low-resolution ESI-MS of Co trefoil knot Δ-**3**. Calculated peaks (m/z): 1894.6263 $[Co^{III}{}_2Co^{II}\text{-}BF_4]^{+}$; 1807.6234 $[Co^{III}Co^{II}{}_2\text{-}BF_4]^{+}$; 947.3131 $[M\text{-}2BF_4]^{2+}$; 903.8111 $[Co^{III}{}_2Co^{II}\text{-}2BF_4]^{2+}$; 602.5411 $[M\text{-}3BF_4]^{3+}$. Partial reduction of the compounds was apparent under the ESI-MS conditions.

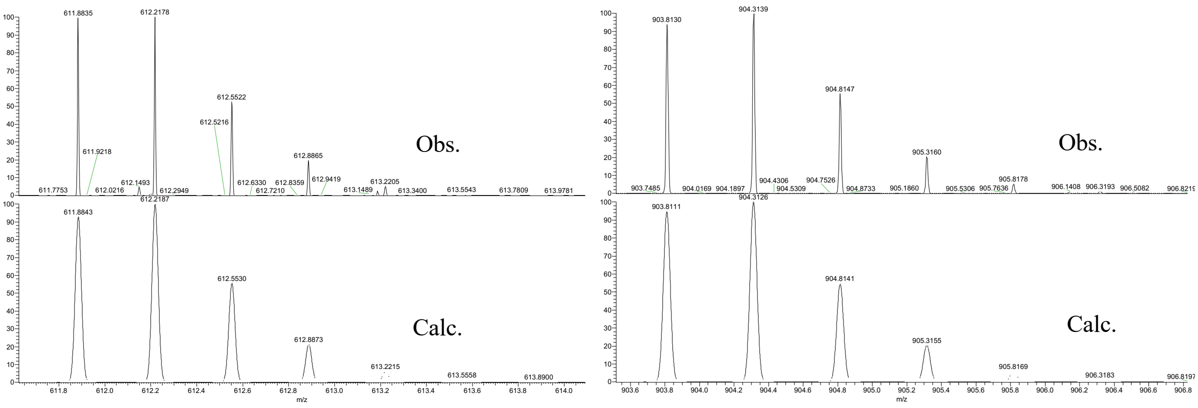

Figure S4: High-resolution ESI-MS of Co trefoil knot Δ-**3**. (a) isotopic distribution of $[M\text{-}3BF_4]^{2+}$ (left); (b) isotopic distribution of $[Co^{III}{}_2Co^{II}\text{-}2BF_4]^{2+}$ (right).

3.3 Synthesis of wholly organic trefoil knot Δ-**4**

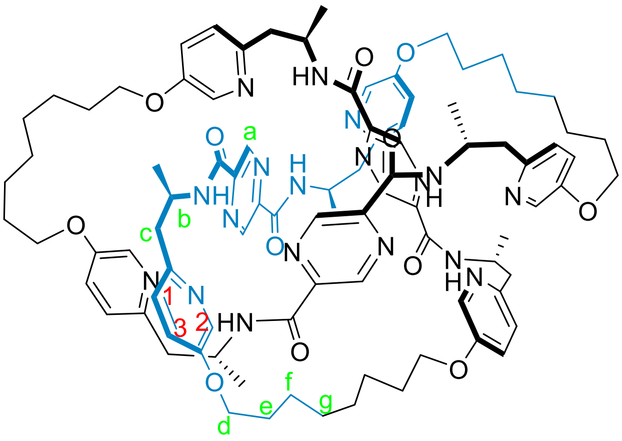

Δ-**4**

Compound Δ-**3** (18 mg, 9.1 μmol) was dissolved in MeOH (10 ml) and acetic acid (10 ml). Activated Zn (117 mg) was added, and the resulting suspension was stirred for 2 h under an atmosphere of air. The solvent was removed under reduced pressure and the crude residue was redissolved in $CHCl_3$ (50 mL) and washed with a 17.5% NH_3 solution saturated with Na_4EDTA (50 mL). The aqueous layer was extracted with $CHCl_3$ (3 x 50 mL). The combined organic layers were washed with brine (50 mL), dried (Na_2SO_4) and concentrated to dryness. The crude mixture was redissolved in CH_2Cl_2 (2 mL) and MeOH (2 ml). Pd/C was added and the resulting suspension was repeatedly degassed and purged with N_2 before being repeatedly degassed and purged with H_2 and stirred at 40 °C for 24 h under an atmosphere of H_2. The reaction mixture was filtered through celite and the solvent was removed under reduced pressure and purified by preparative thin layer chromatography (98:2 CH_2Cl_2: MeOH as eluent) to yield Δ-**4** as a colourless solid (5.2 mg, 35%). ^{1}H NMR (600 MHz, $CDCl_3$) δ 8.88 (s, 6H, H_a), 8.03 (d, J = 7.9 Hz, 6H, NH), 7.80 (d, J = 2.7 Hz, 6H, H_2), 6.99 (d, J = 8.5 Hz, 6H, H_3), 6.61 – 6.46 (m, 6H, H_1), 4.54 – 4.42 (m, 6H, H_b), 3.30 – 3.07 (m, 18H, $H_{c,d}$), 2.87 – 2.80 (m, 6H, $H_{c'}$), 1.44 (d, J = 6.4 Hz, 18H, H_{Me}), 1.13 – 1.07 (m, 12H, H_e), 0.85 – 0.64 (m, 24H, $H_{f,g}$). ^{13}C NMR (151 MHz, $CDCl_3$) δ 161.52, 153.36, 150.46, 145.91, 141.71, 137.01, 123.32, 120.84, 67.27, 46.81, 43.51, 29.86, 28.75, 25.18, 21.93.

HR ESI-MS: 820.9530 $[M+2H]^{2+}$ (calcd: 820.9521 for $C_{90}H_{116}N_{18}O_{12}$)

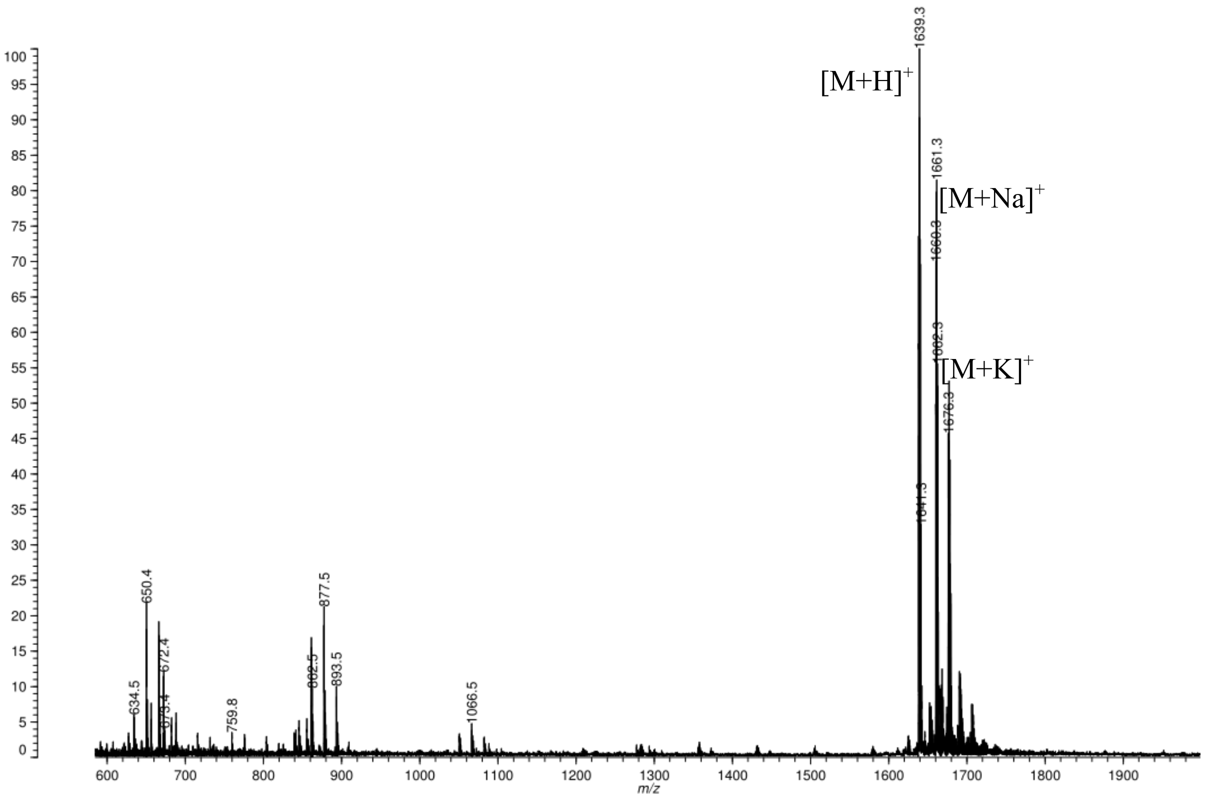

Figure S5: MALDI-MS of the organic knot Δ-**4**.

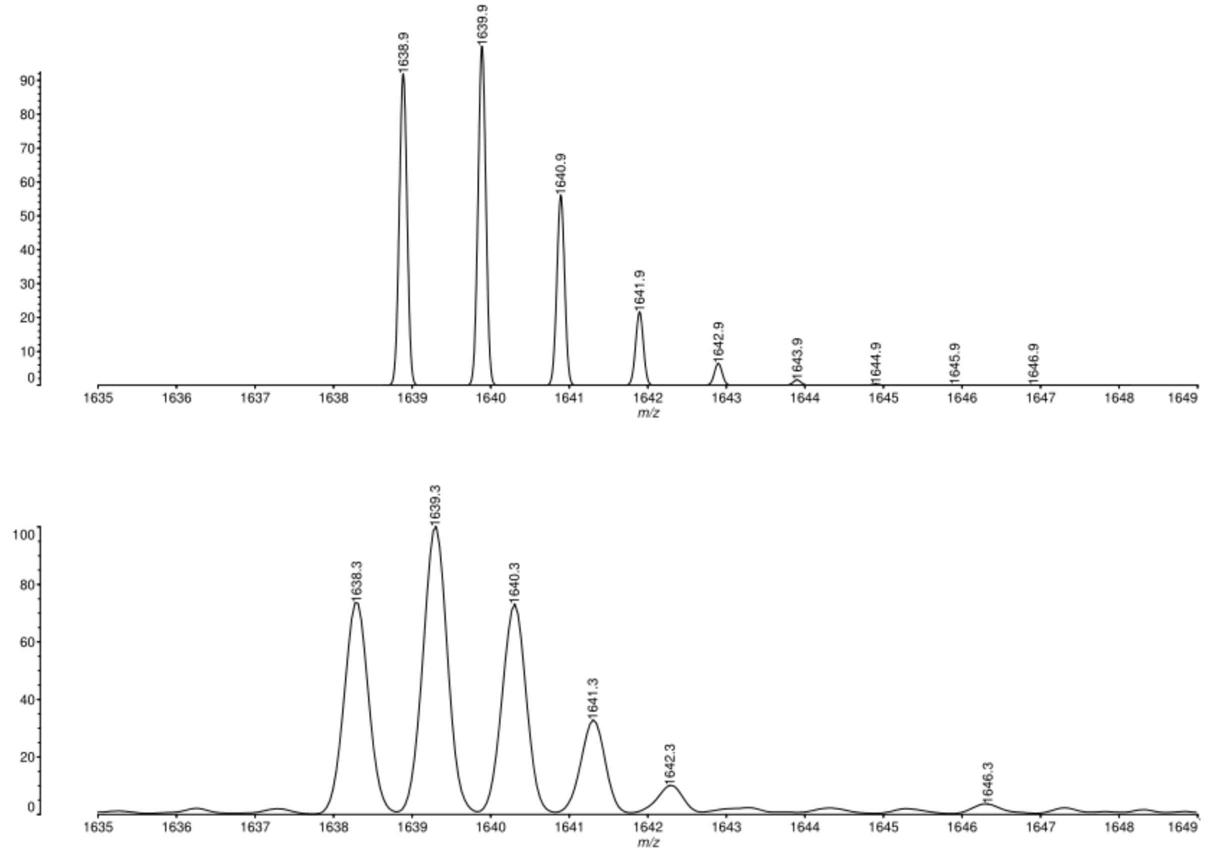

Figure S6: ESI-MS of the organic knot Δ-**4**, with predicted isotopic distribution of $[M+H]^+$.

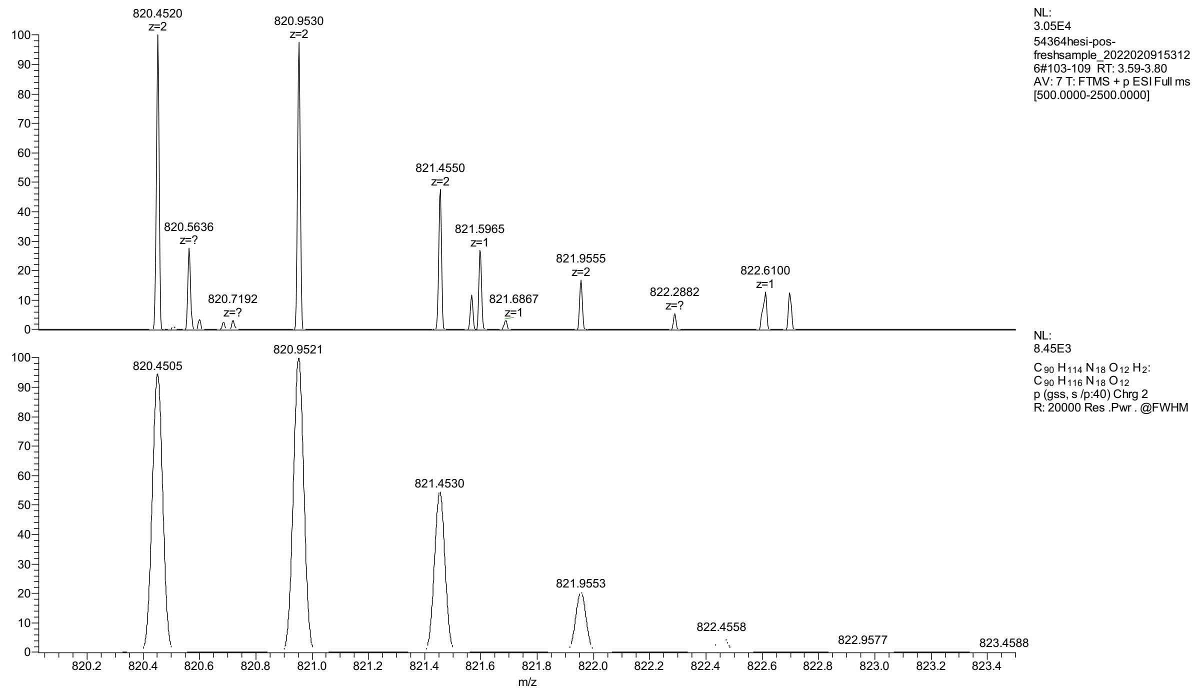

Figure S7: High-resolution ESI-MS of the organic knot **Δ-4**, with predicted isotopic distribution of $[M+2H]^{2+}$. Experimental spectrum (top) and calculated spectrum (bottom).

4. CD and UV/Vis Spectra

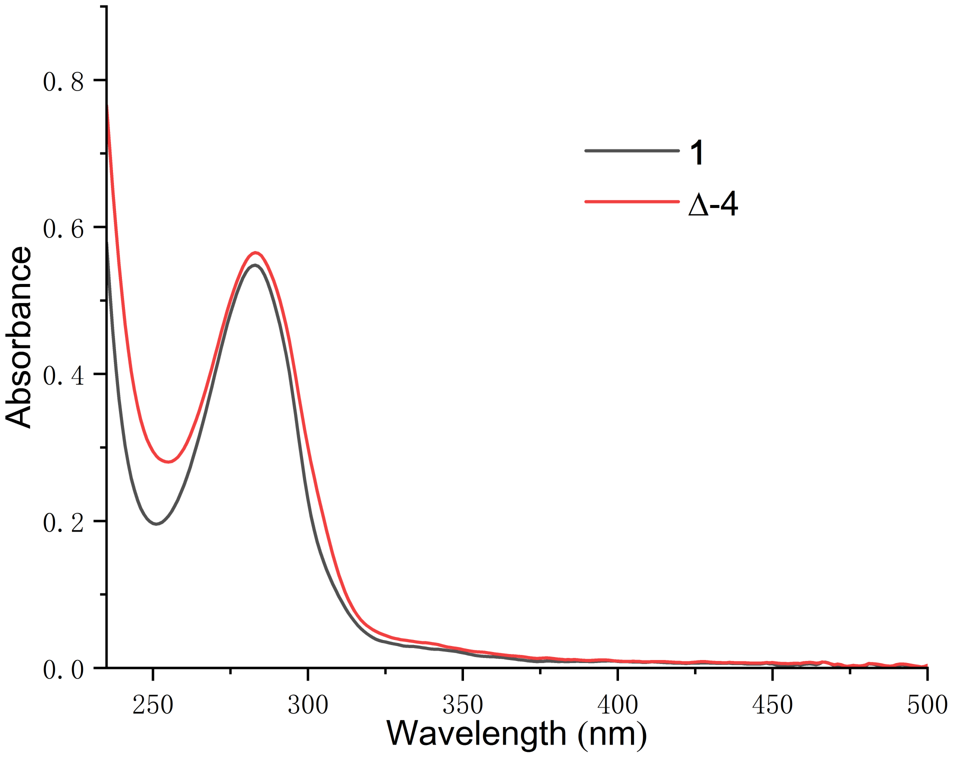

Figure S8: UV/Vis spectra of **1** and Δ-**4** (0.05 mM, CH_2Cl_2, 298K).

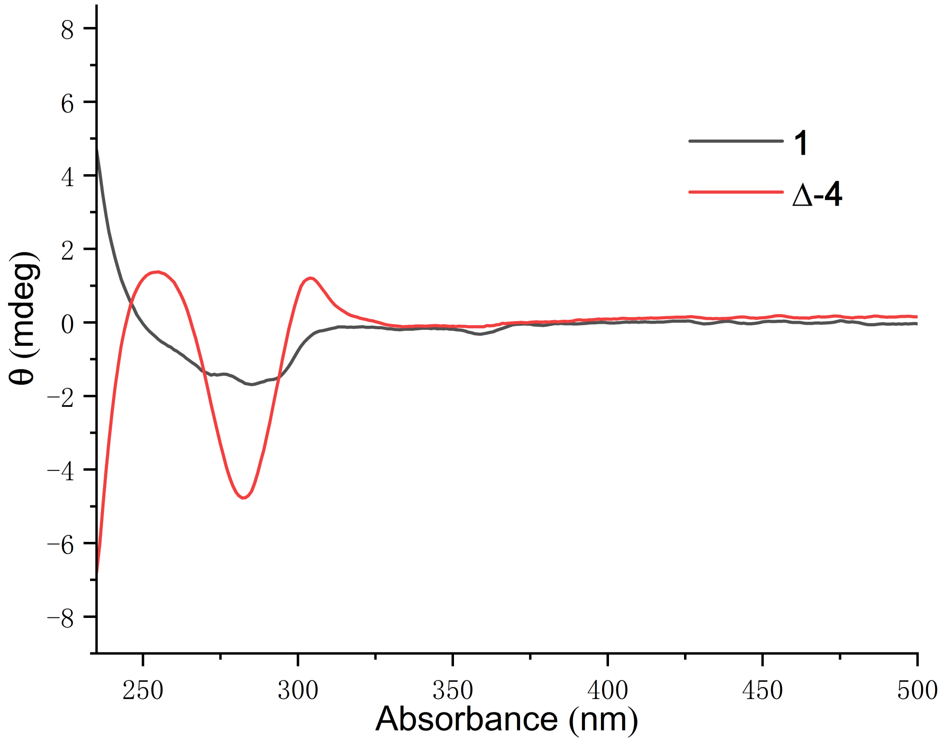

Figure S9: CD spectra of **1** and Δ-**4** (0.05 mM, CH_2Cl_2, 298K).

5. X-ray Crystal Structure Information

Data Collection. X-Ray Data for the overhand knot Δ-**2**·[Co_3] and the corresponding trefoil knot Δ-**3**·[Co_3] were collected at a temperature of 100 K with Zr-L radiation (λ = 0.6889) using the synchrotron X-Ray source at single crystal X-Ray diffraction beamline I19 in Diamond light Source,[S4] equipped with an Pilatus 2M detector and an Oxford Cryosystems Cryostream nitrogen flow gas system using GDA suite of programs. Data for Δ-**2**·[Co_3] and Δ-**3**·[Co_3] were reduced using CrysAlisPro v171.41.xx and absorption correction was performed using empirical methods (SCALE3 ABSPACK) based upon symmetry-equivalent reflections combined with measurements at different azimuthal angles.[S5] The structures for both were solved and refined against all F^2 values using Shelx-2018/3 implemented through Olex2 v1.3 and v1.5.[S6]

In both cases the structures were highly disordered. Appropriate 1,2- and 1,3- fixed distance and same distance restraints were applied to the relevant bonding motifs. Strong similar neighbouring atomic displacement parameters and rigid bond restraints were applied globally to account for the close overlap of refined atomic sites. Appropriate occupancies were fixed to match the expected occupancies for the different terminal and bridging groups. Solvent masks were applied to both structures to account for the contribution of the electron density within the poorly defined solvent voids to the observed intensities.

Table S1. Crystallographic information for samples Δ-**2**·[Co_3] and Δ-**3**·[Co_3]

Identification code	Δ-**2**·[Co_3]	Δ-**3**·[Co_3]
Empirical formula	$C_{92}H_{110}B_3Co_3F_{12}N_{18}O_{12}$	$C_{90}H_{106}B_3Co_3F_{12}N_{18}O_{12}$
Formula weight	2097.19	2069.14
Temperature/K	100.00(10)	100.00(10)
Crystal system	trigonal	trigonal
Space group	P321	P321
a/Å	19.2649(12)	19.1834(5)
b/Å	19.2649(12)	19.1834(5)
c/Å	10.4039(4)	10.3773(2)
α/°	90	90.0
β/°	90	90.0
γ/°	120	120.0
Volume/$Å^3$	3344.0(4)	3307.24(18)
Z	1	1
$\rho_{calc}g/cm^3$	1.041	1.039
μ/mm^{-1}	0.402	0.406
F(000)	1088.0	1072.0
Crystal size/mm^3	0.085 × 0.065 × 0.055	0.08 × 0.06 × 0.05
Radiation	synchrotron (λ = 0.6889)	synchrotron (λ = 0.6889)
2Θ range for data collection/°	4.098 to 48.972	3.804 to 51.694
Index ranges	$-23 \le h \le 23$, $-23 \le k \le 23$, $-12 \le l \le 12$	$-24 \le h \le 24$, $-24 \le k \le 24$, $-13 \le l \le 13$
Reflections collected	37351	45032
Independent reflections	4091 [R_{int} = 0.1253, R_{sigma} = 0.0456]	4696 [R_{int} = 0.0446, R_{sigma} = 0.0266]
Data/restraints/parameters	4091/784/406	4696/1650/561
Goodness-of-fit on F^2	1.212	1.011
Final R indexes [I>=2σ (I)]	R_1 = 0.1162, wR_2 = 0.2982	R_1 = 0.0716, wR_2 = 0.2089
Final R indexes [all data]	R_1 = 0.1329, wR_2 = 0.3197	R_1 = 0.0820, wR_2 = 0.2255
Largest diff. peak/hole / e $Å^{-3}$	1.01/-0.47	0.55/-0.37
Flack parameter	0.09(2)	0.046(12)

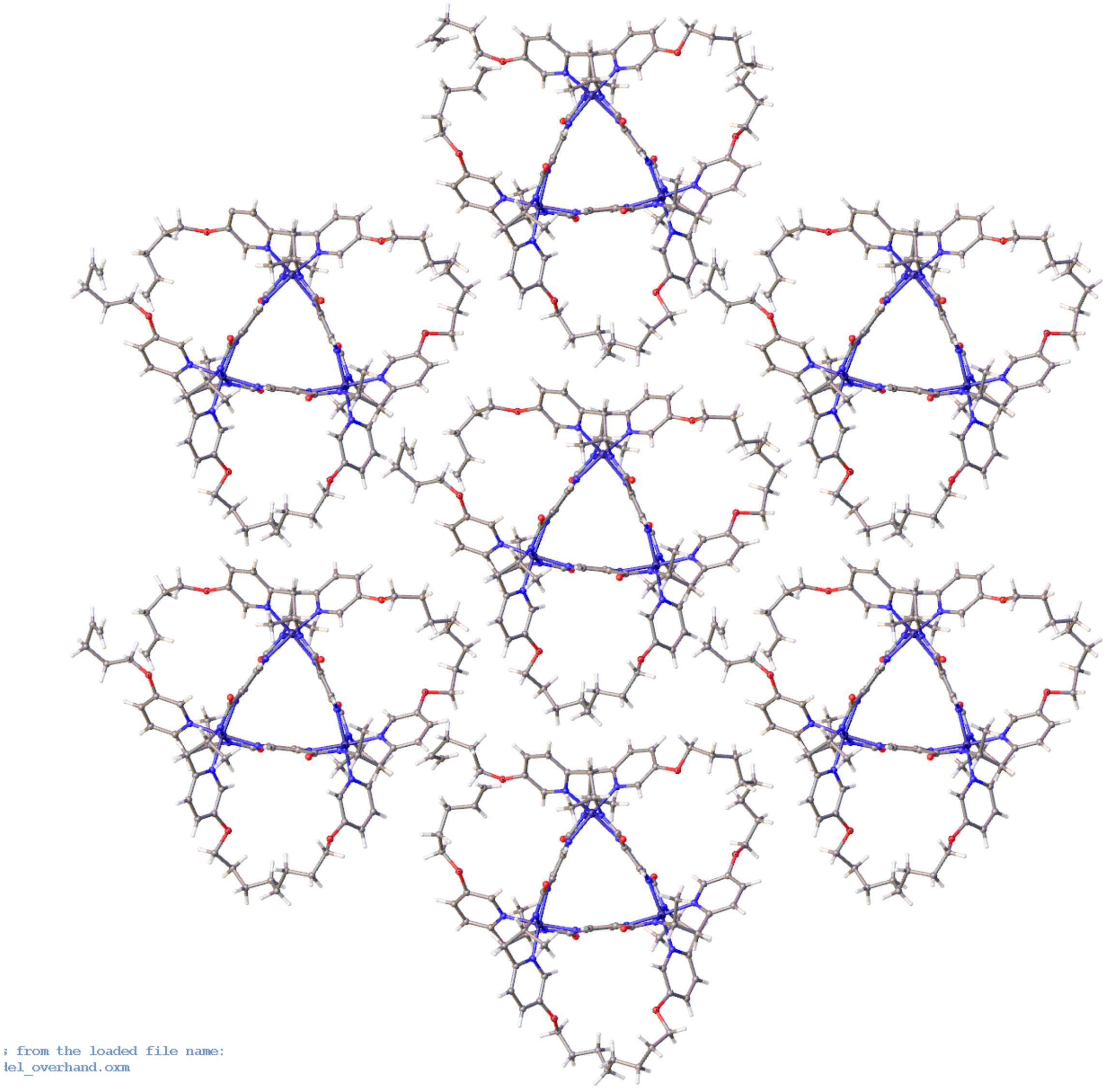

Figure S10. Extended crystal structure of overhand knot Δ-**2**•[Co_3].

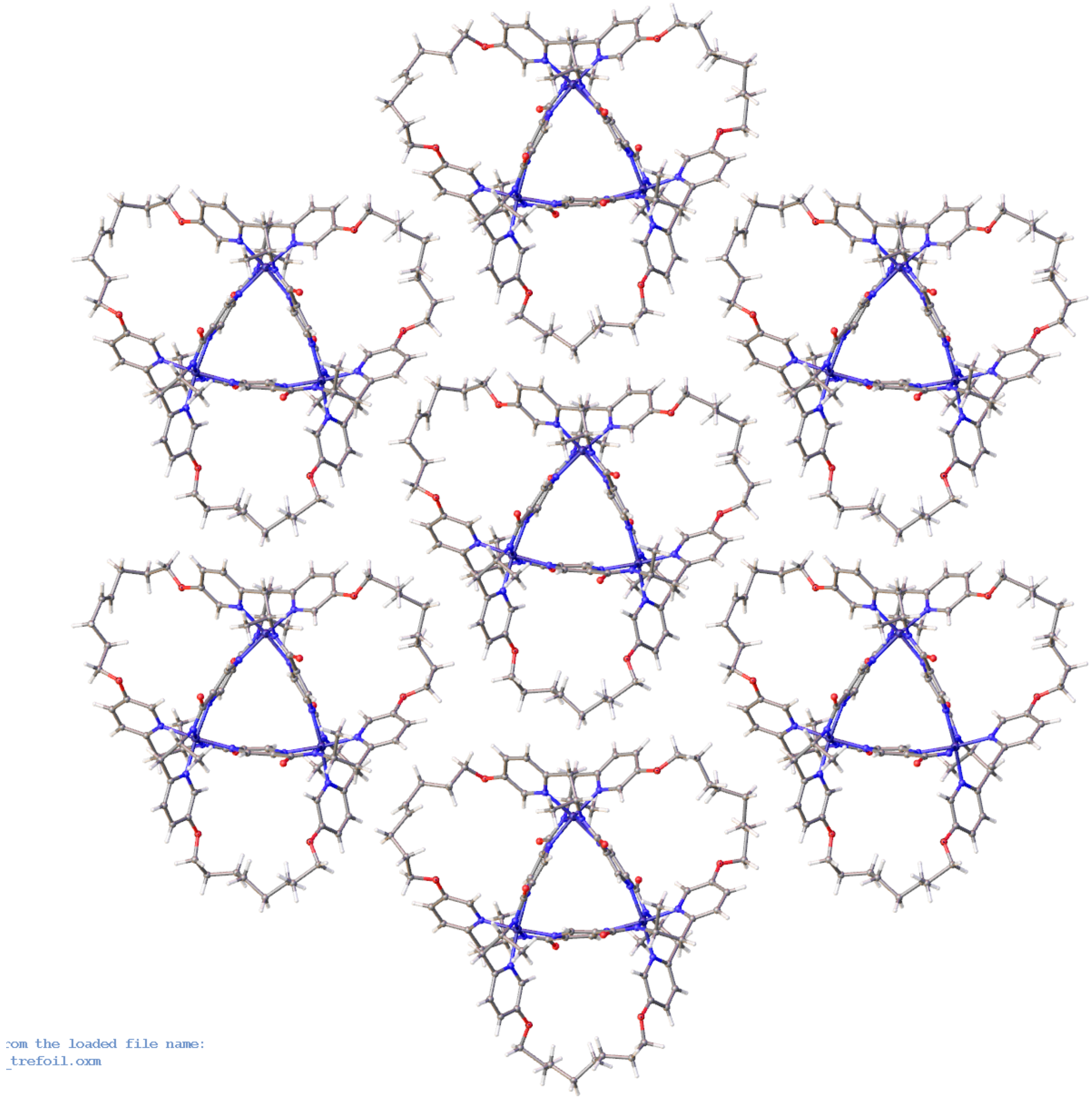

Figure S11. Extended crystal structure of trefoil knot Δ-**3**•[Co_3]

6. NMR Spectra

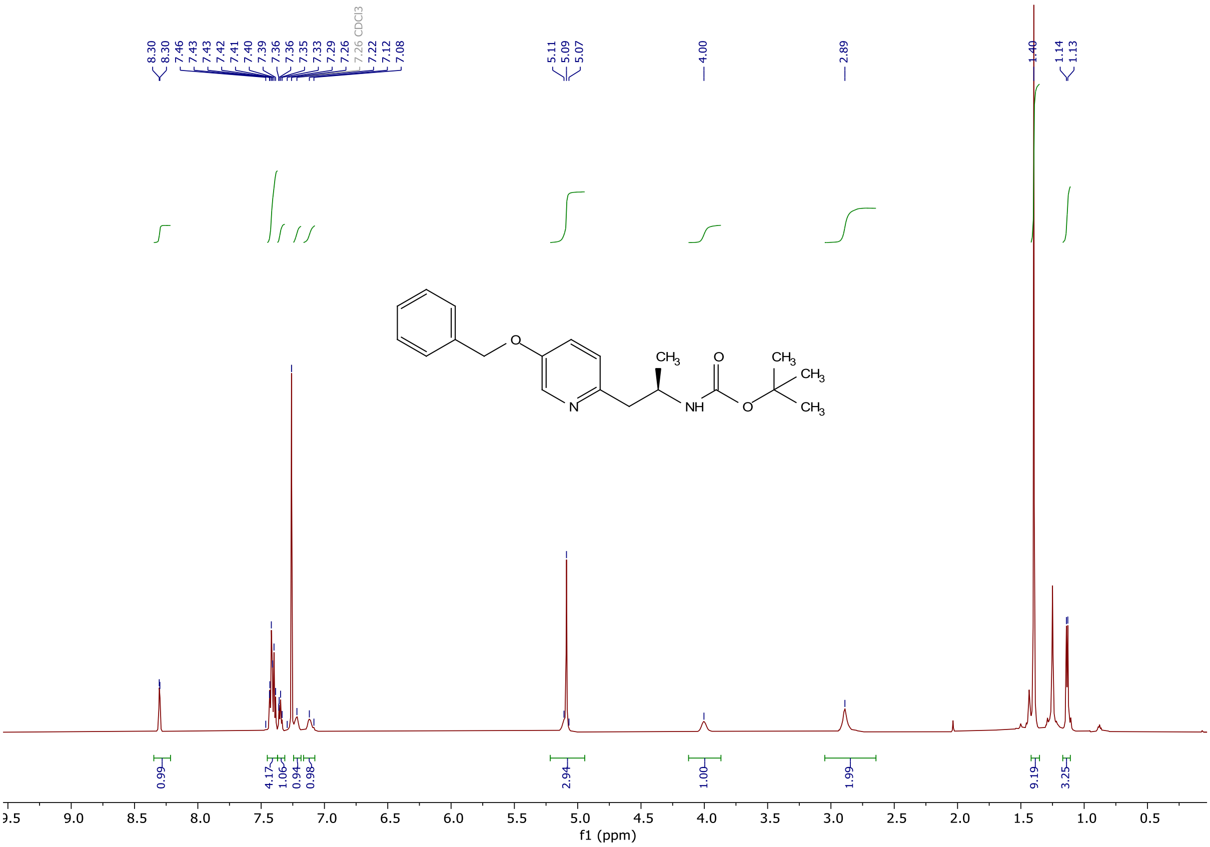

Figure S12: ^{1}H NMR (600 MHz, 298 K, $CDCl_3$) of **6**.

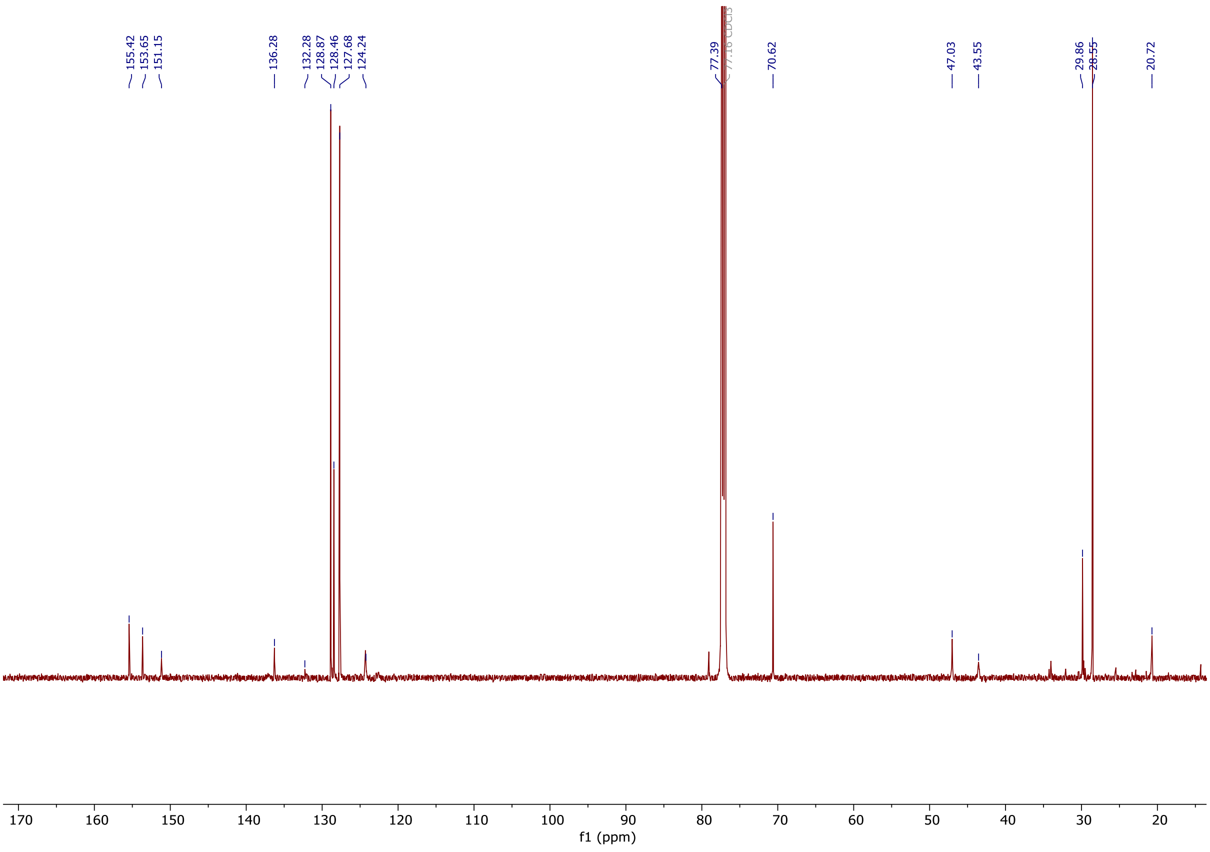

Figure S13: ^{13}C NMR (151 MHz, 298 K, $CDCl_3$) of **6**.

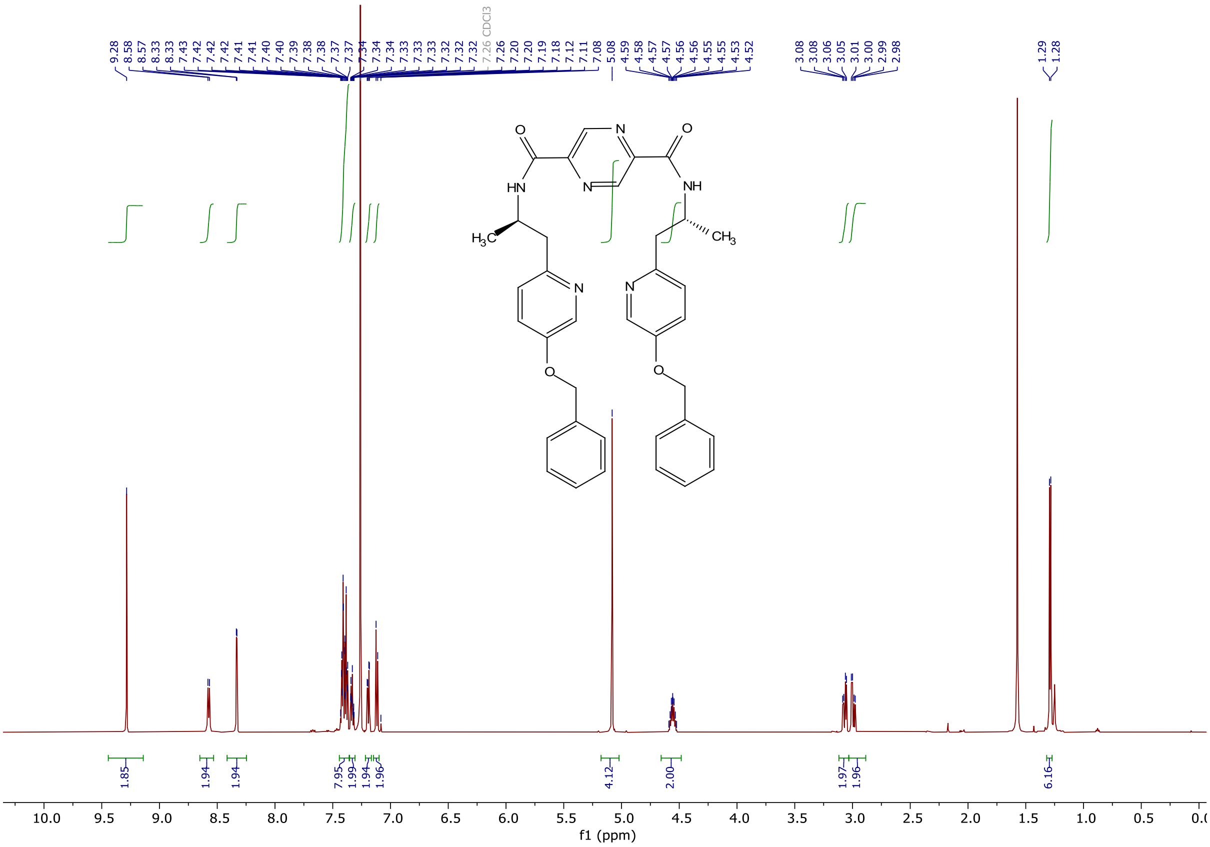

Figure S14: ^{1}H NMR (600 MHz, 298 K, $CDCl_3$) of **7**.

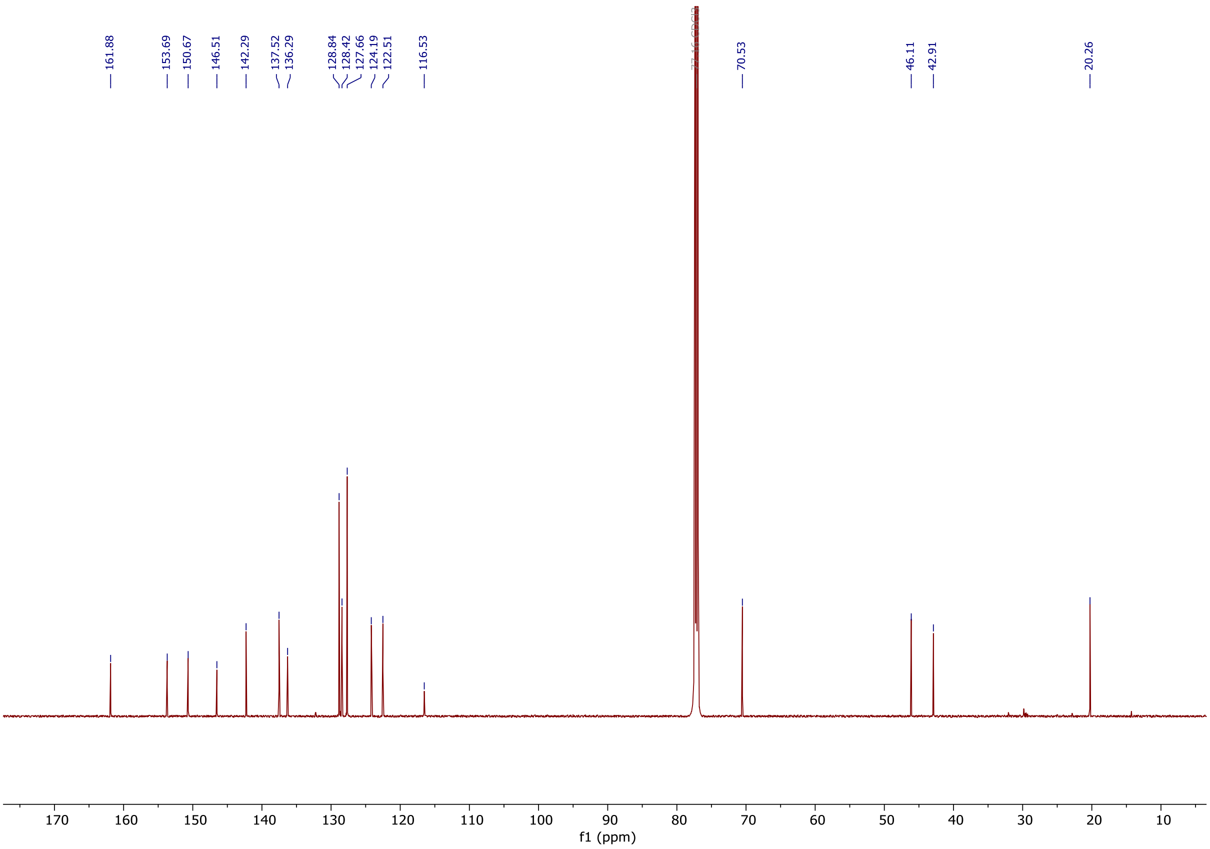

Figure S15: ^{13}C NMR (151 MHz, 298 K, $CDCl_3$) of **7**.

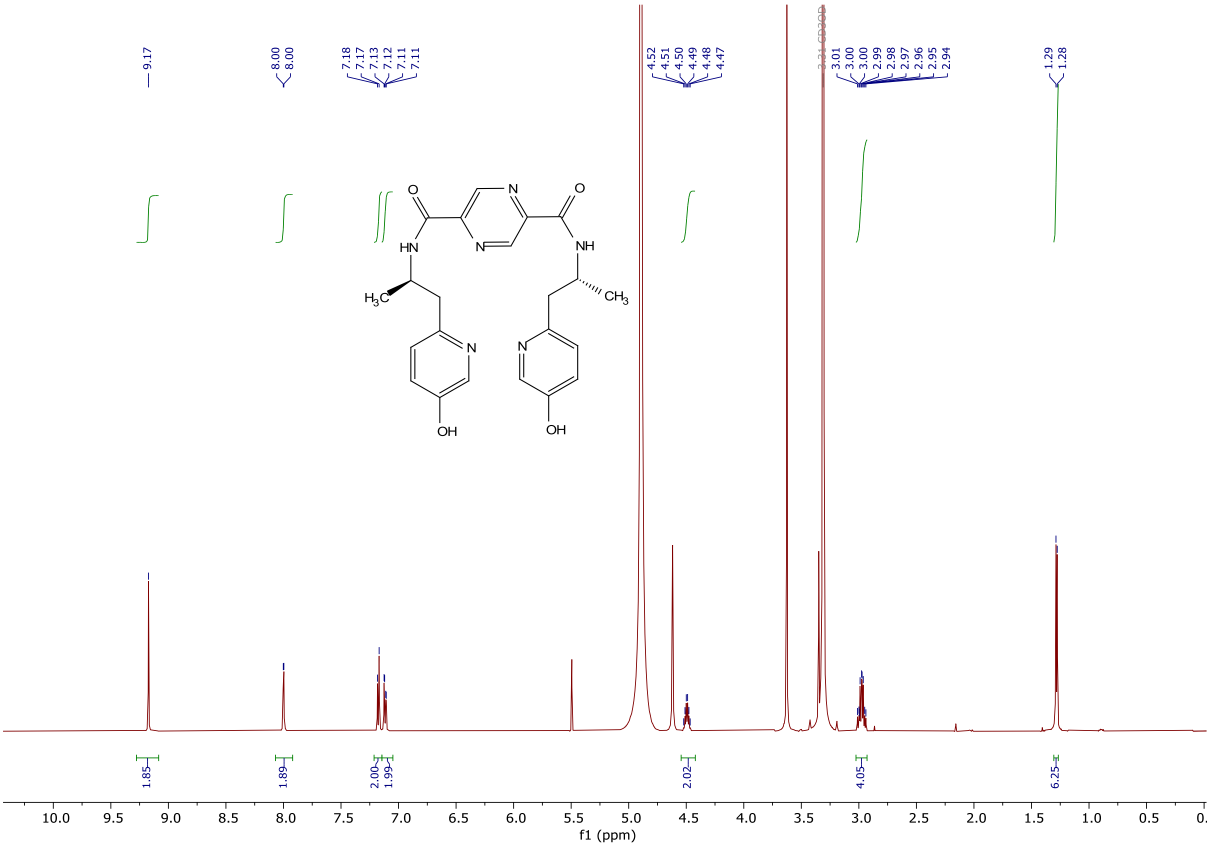

Figure S16: ^{1}H NMR (600 MHz, 298 K, CD_3OD) of **8**.

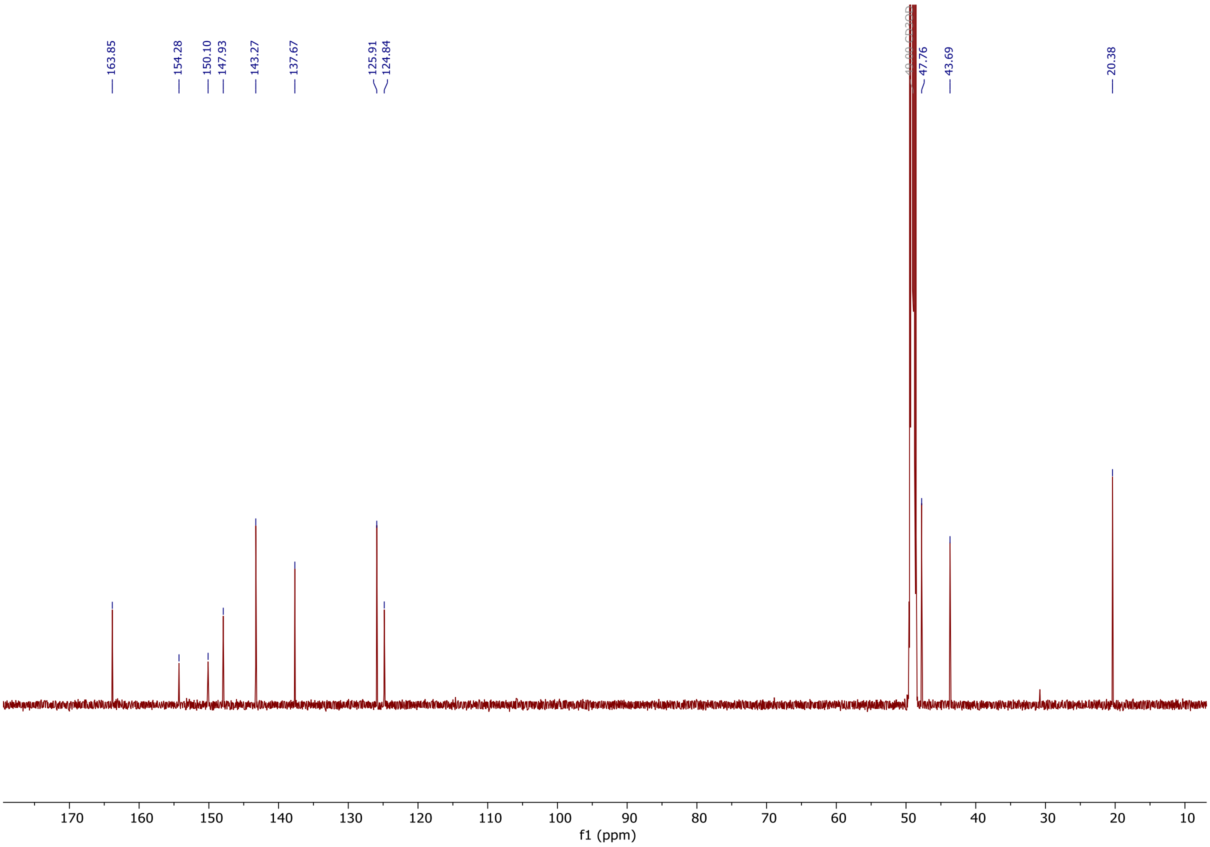

Figure S17: ^{13}C NMR (151 MHz, 298 K, CD_3OD) of **8**.

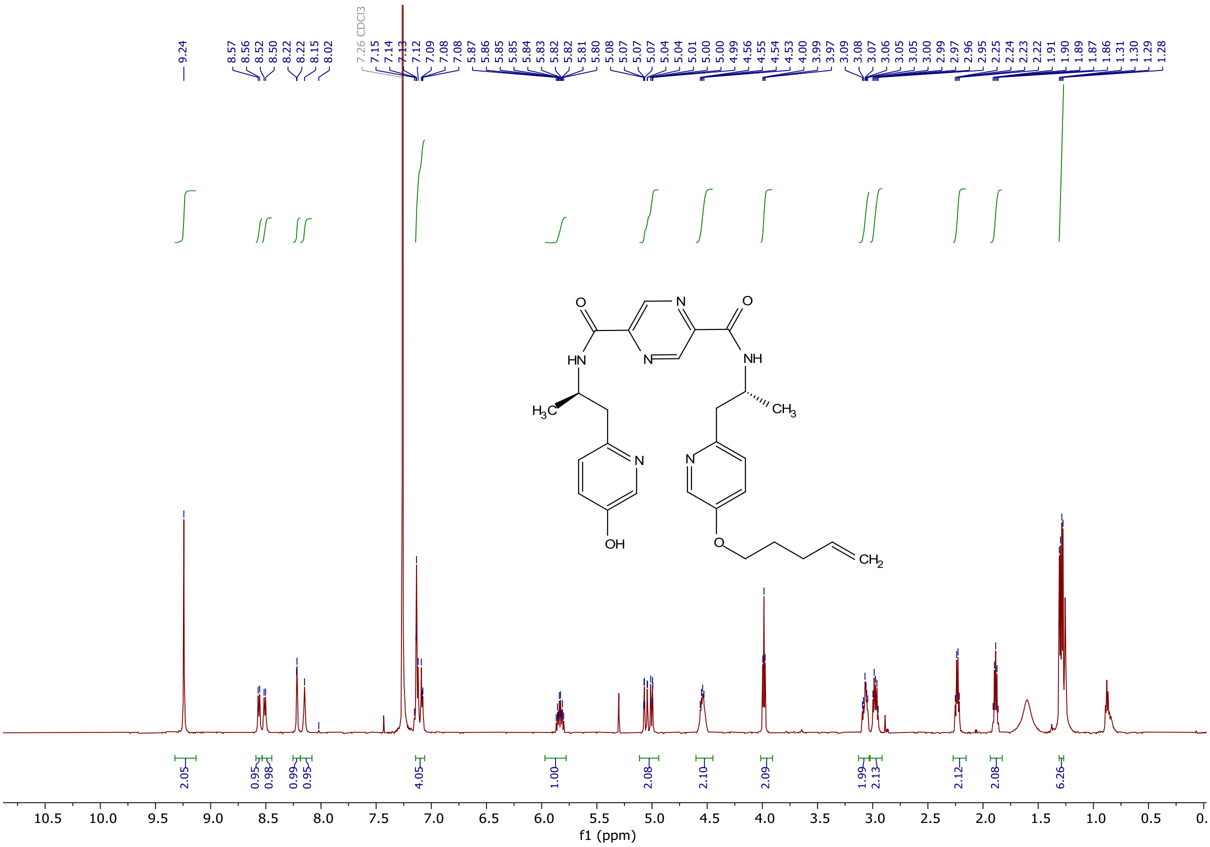

Figure S18: ^{1}H NMR (600 MHz, 298 K, $CDCl_3$) of **9**.

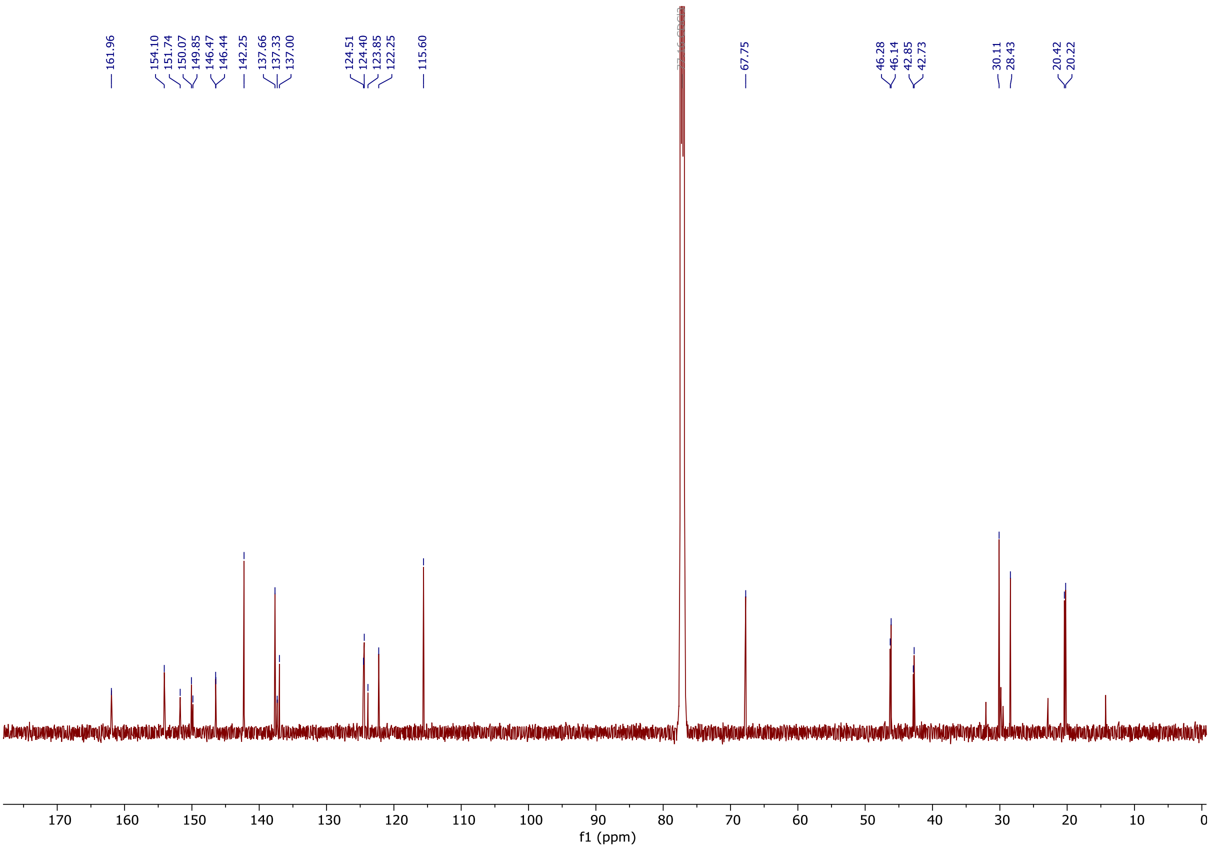

Figure S19: ^{13}C NMR (151 MHz, 298 K, $CDCl_3$) of **9**.

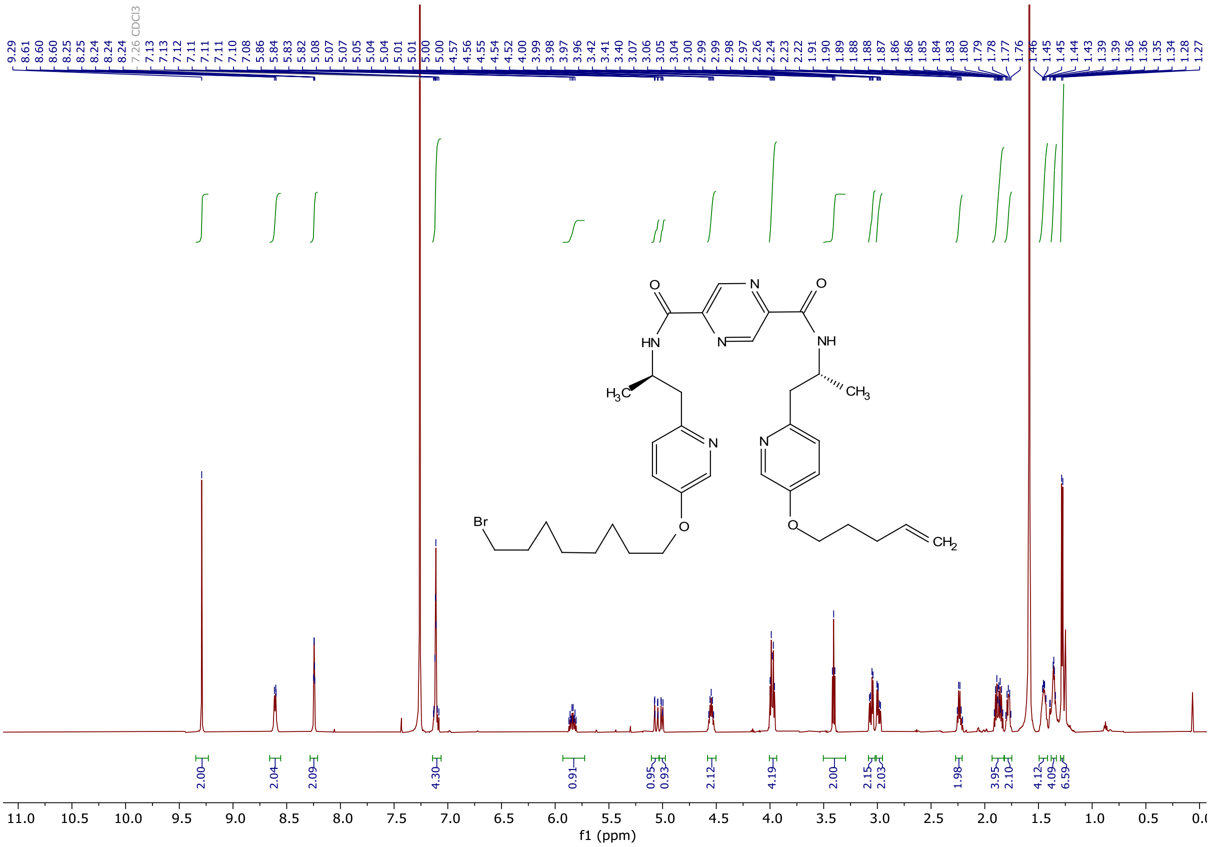

Figure S20: ^{1}H NMR (600 MHz, 298 K, $CDCl_3$) of **10**.

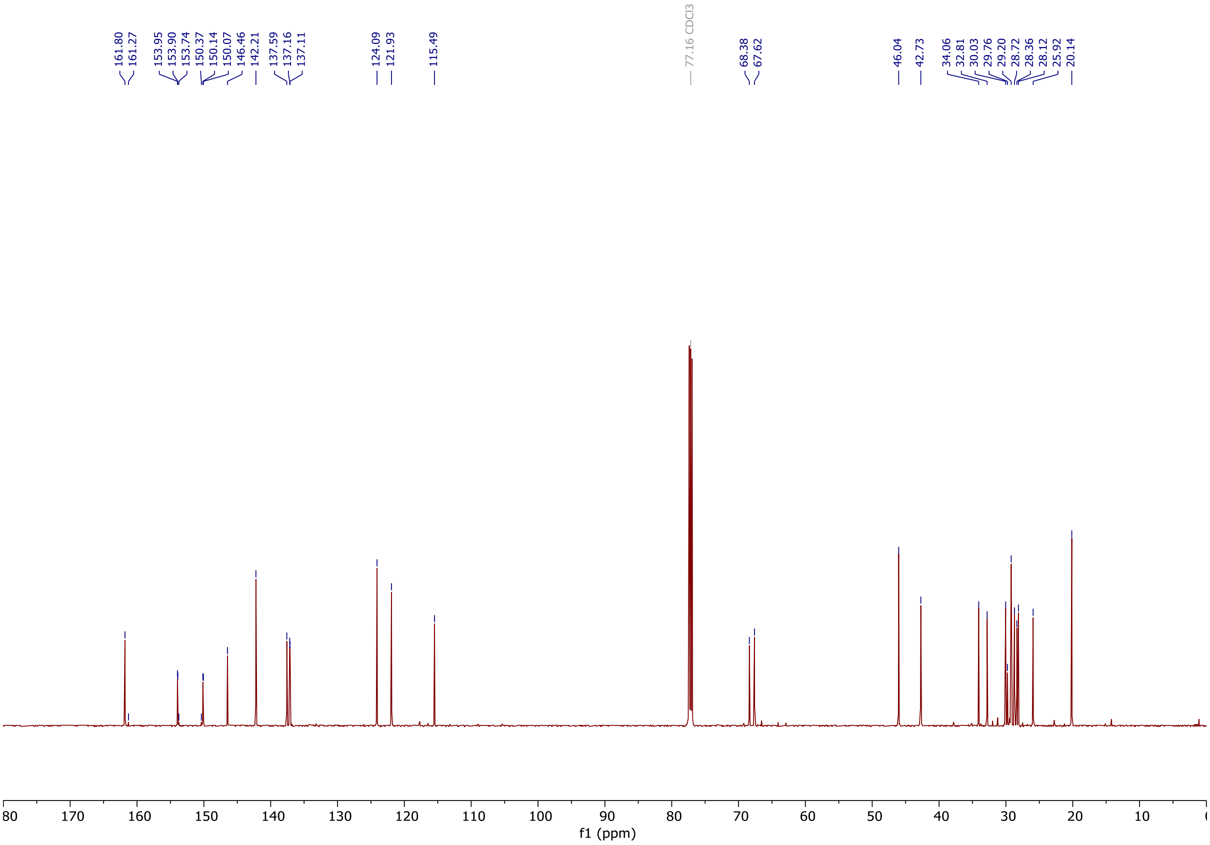

Figure S21: ^{13}C NMR (151 MHz, 298 K, $CDCl_3$) of **10**.

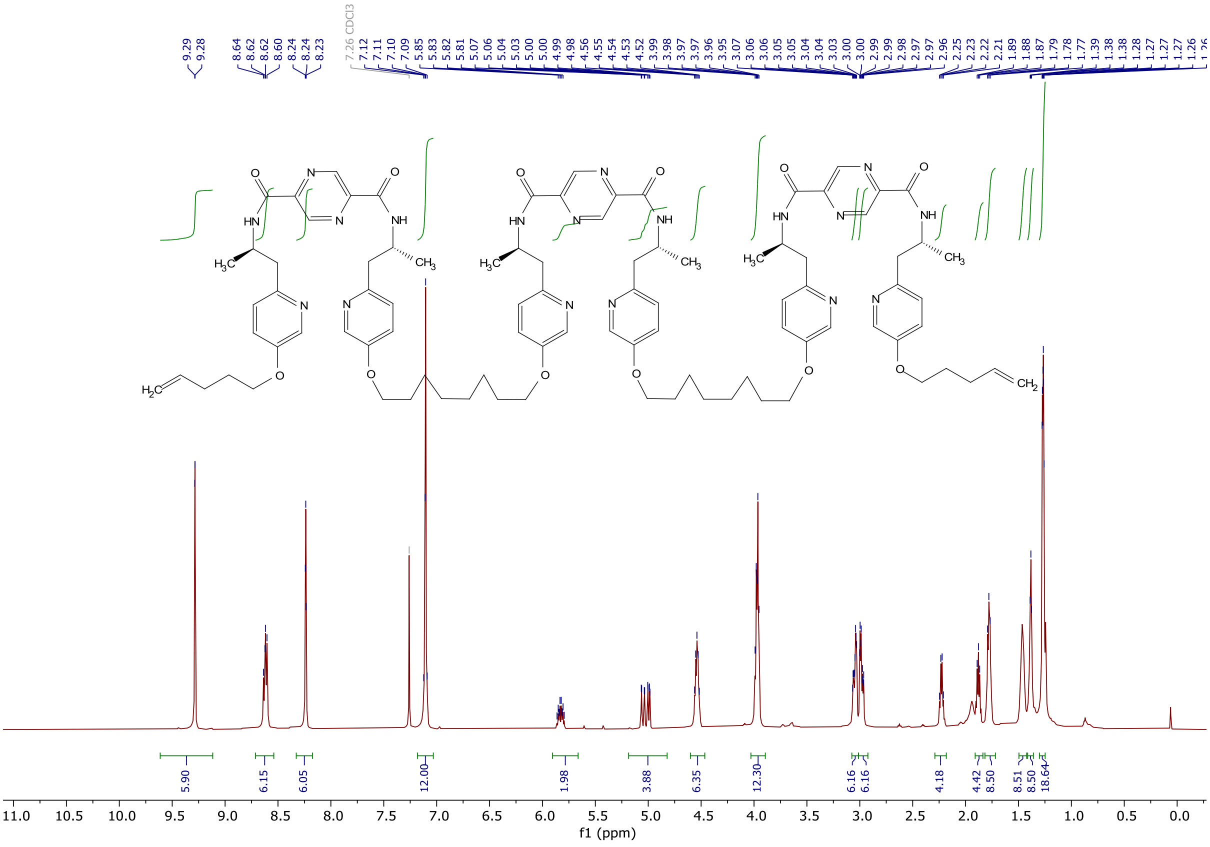

Figure S22: ^{1}H NMR (600 MHz, 298 K, $CDCl_3$) of **1**.

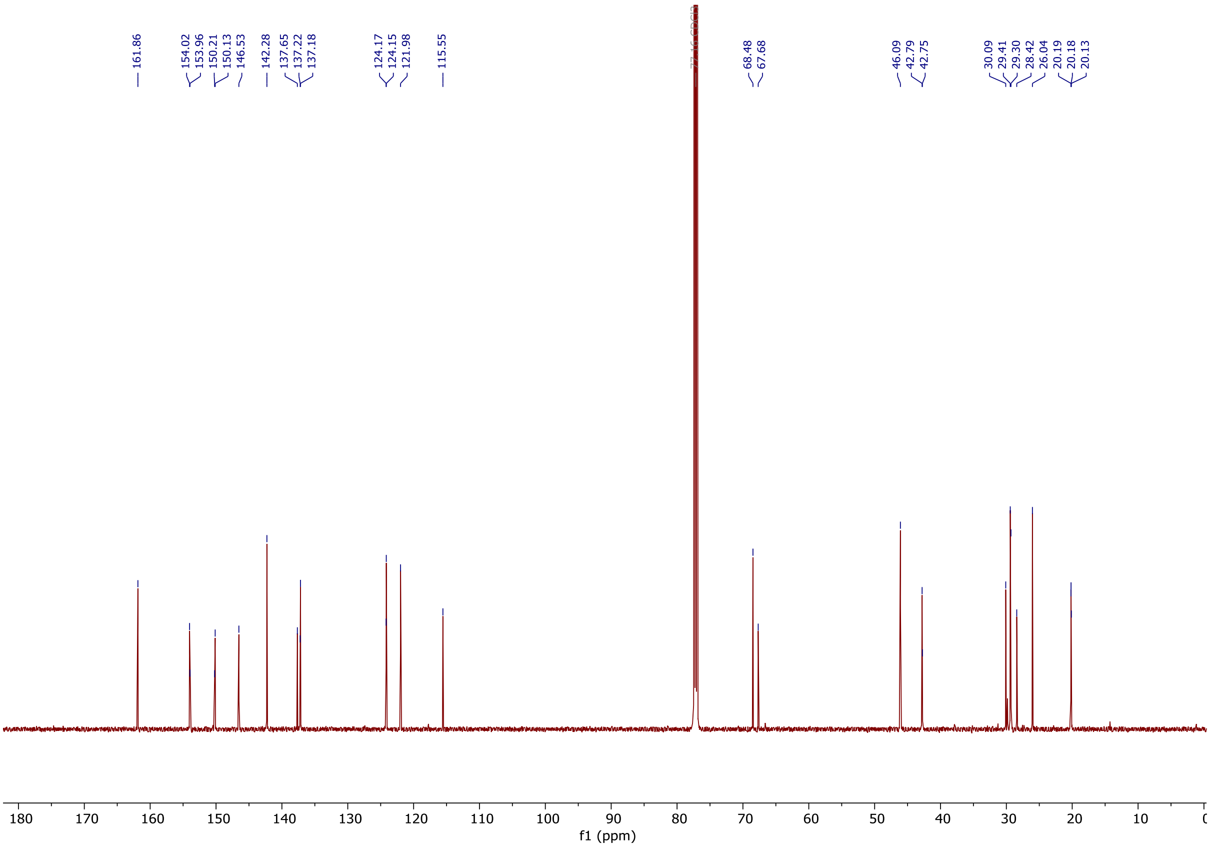

Figure S23: ^{13}C NMR (151 MHz, 298 K, $CDCl_3$) of **1**.

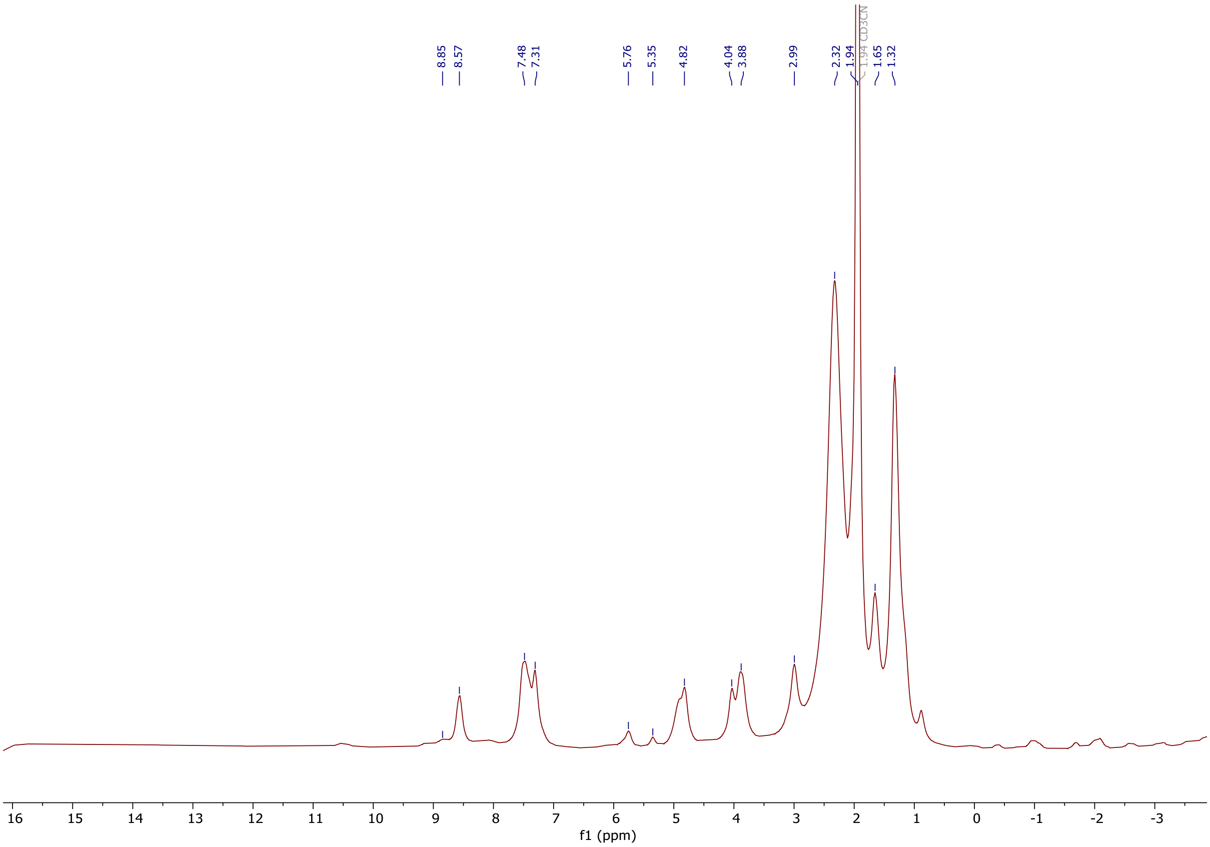

Figure S24: ^{1}H NMR (600 MHz, 298 K, CD_3CN) of overhand trefoil knot Δ-**2**.

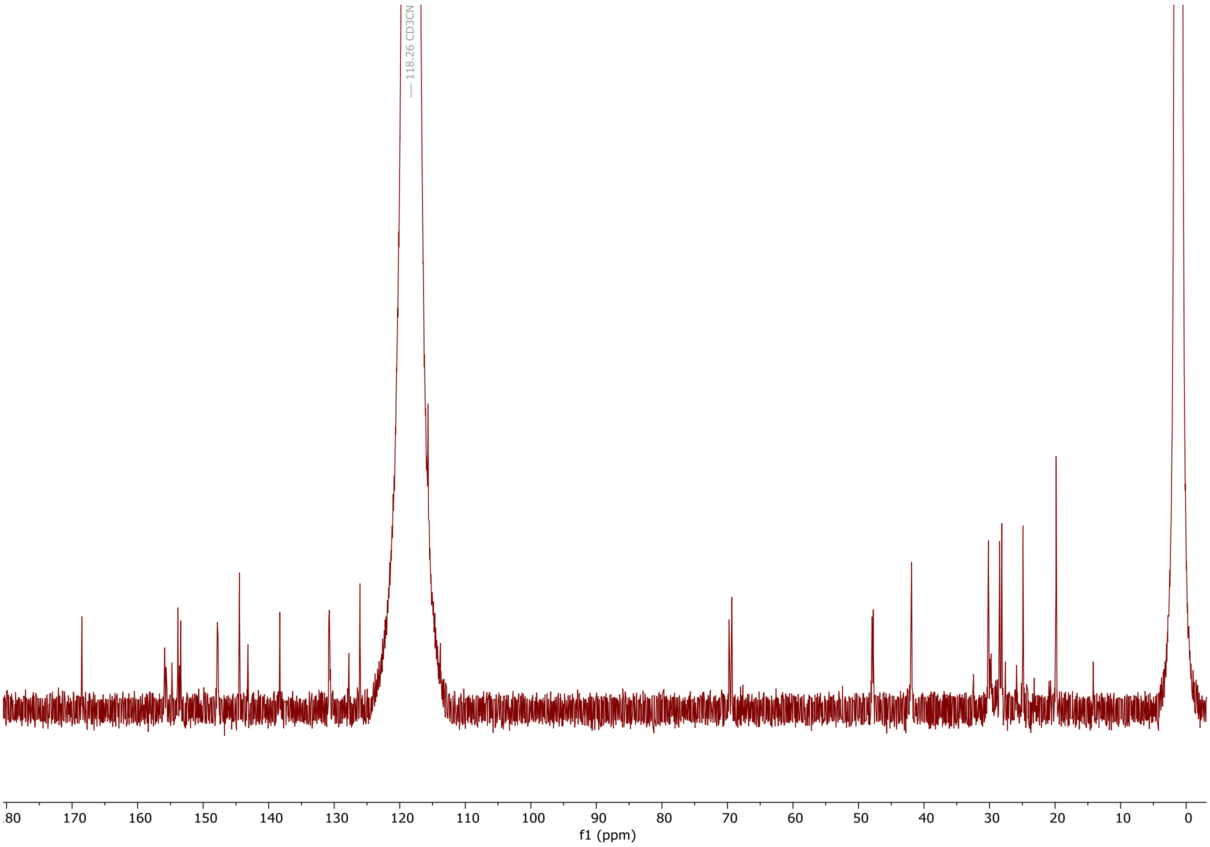

Figure S25: ^{13}C NMR (151 MHz, 298 K, CD_3CN) of overhand trefoil knot Δ-**2**.

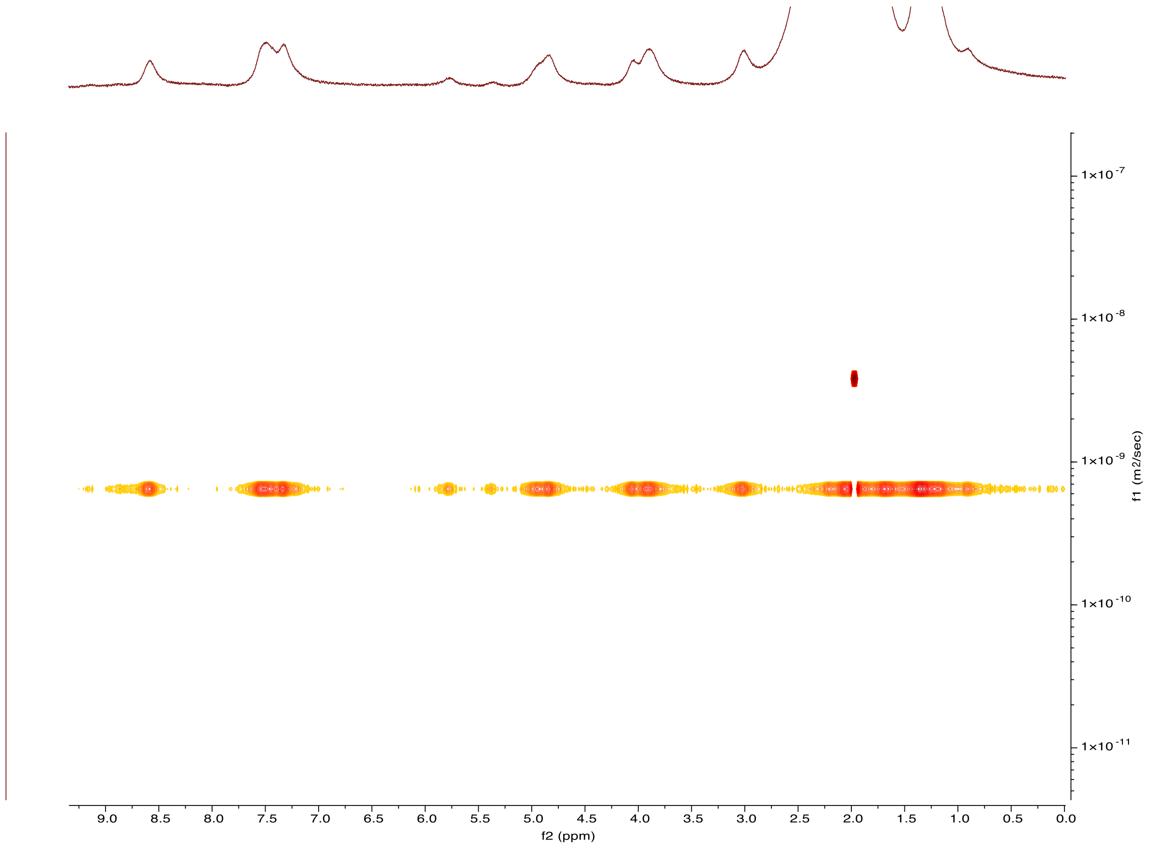

Figure S26: DOSY-^{1}H NMR (600 MHz, 298 K, CD_3CN) of overhand trefoil knot Δ-**2**.

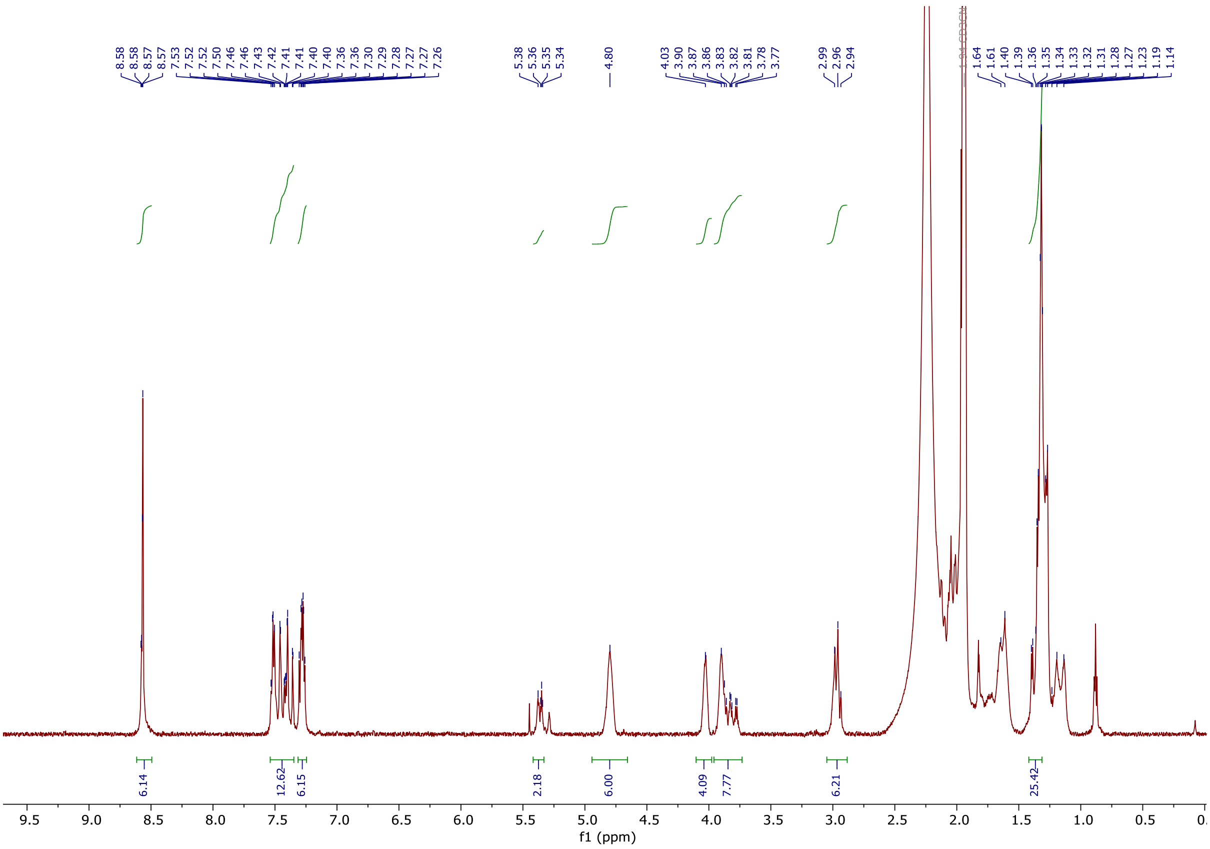

Figure S27: ^{1}H NMR (600 MHz, 298 K, CD_3CN) of trefoil knot Δ-**3**.

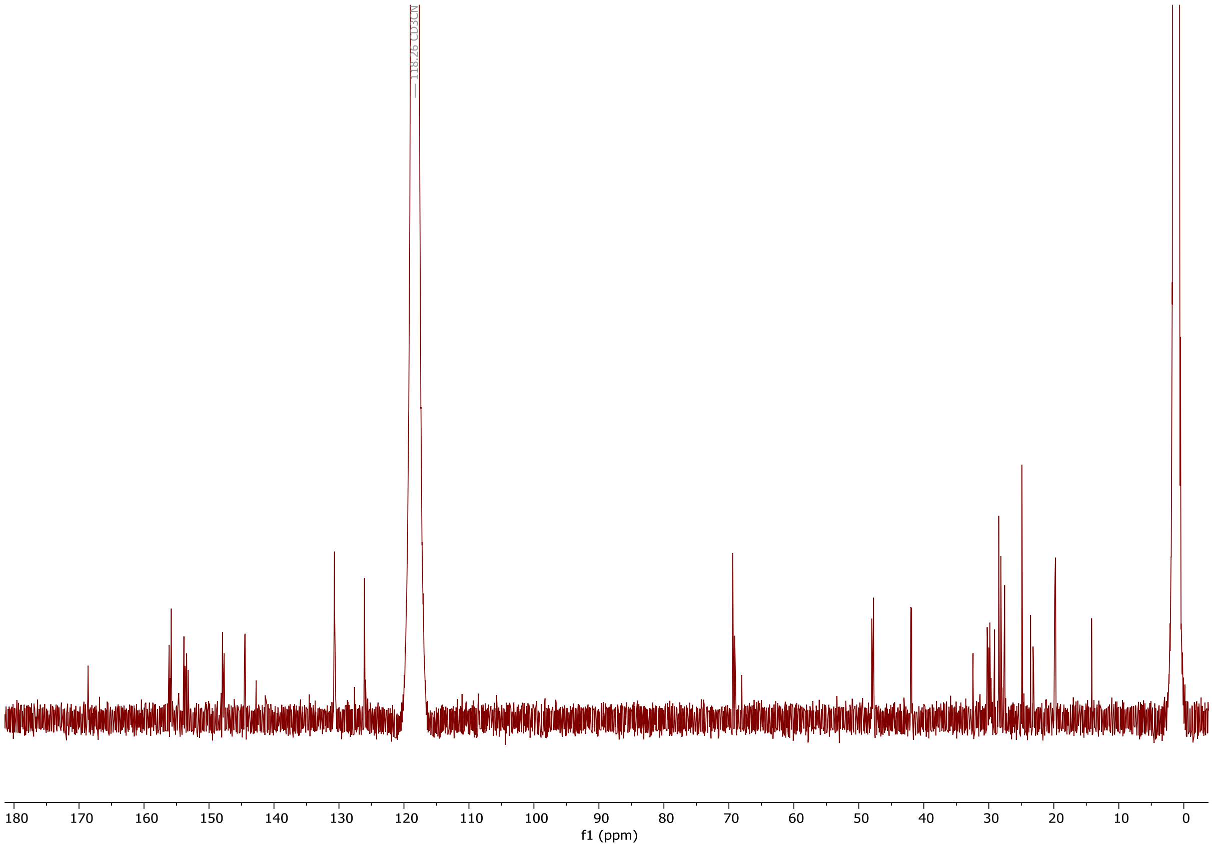

Figure S28: ^{13}C NMR (600 MHz, 298 K, CD_3CN) of trefoil knot Δ-**3**.

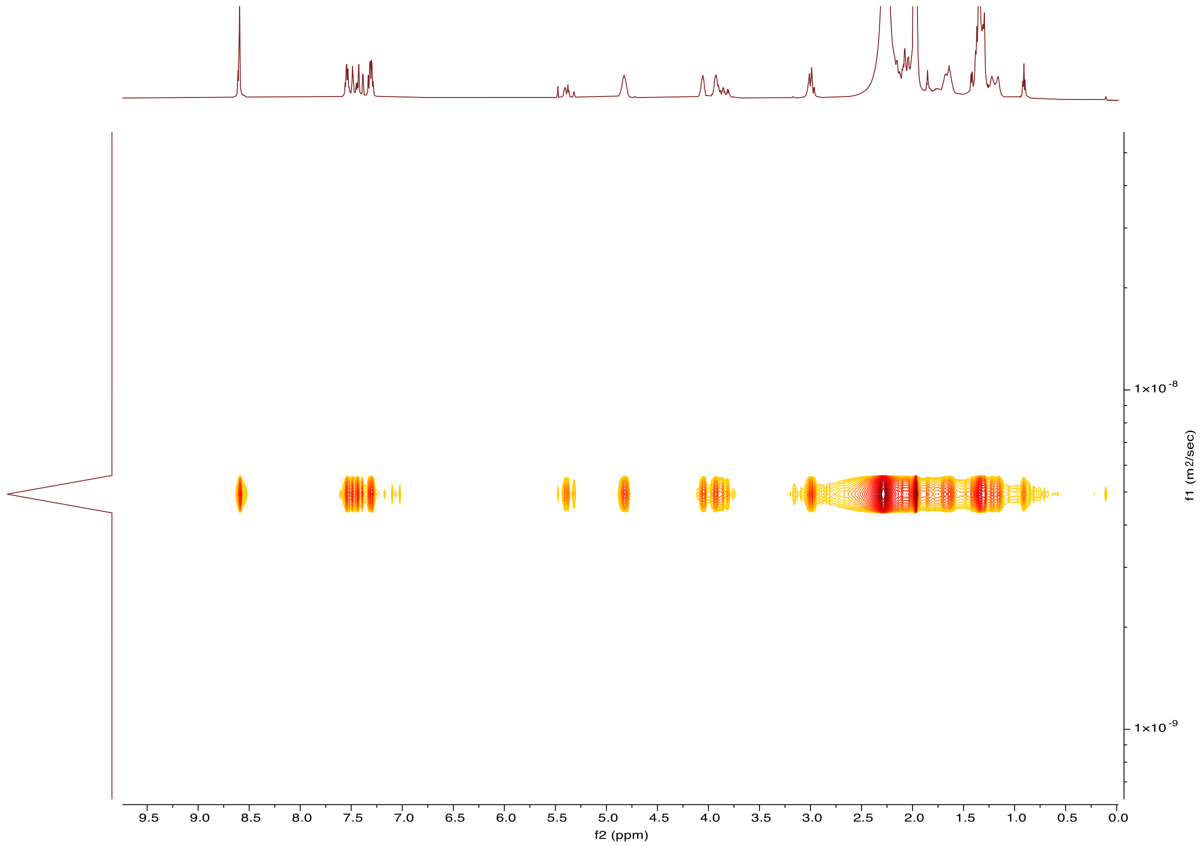

Figure S29: DOSY-^{1}H NMR (600 MHz, 298 K, CD_3CN) of trefoil knot Δ-**3**.

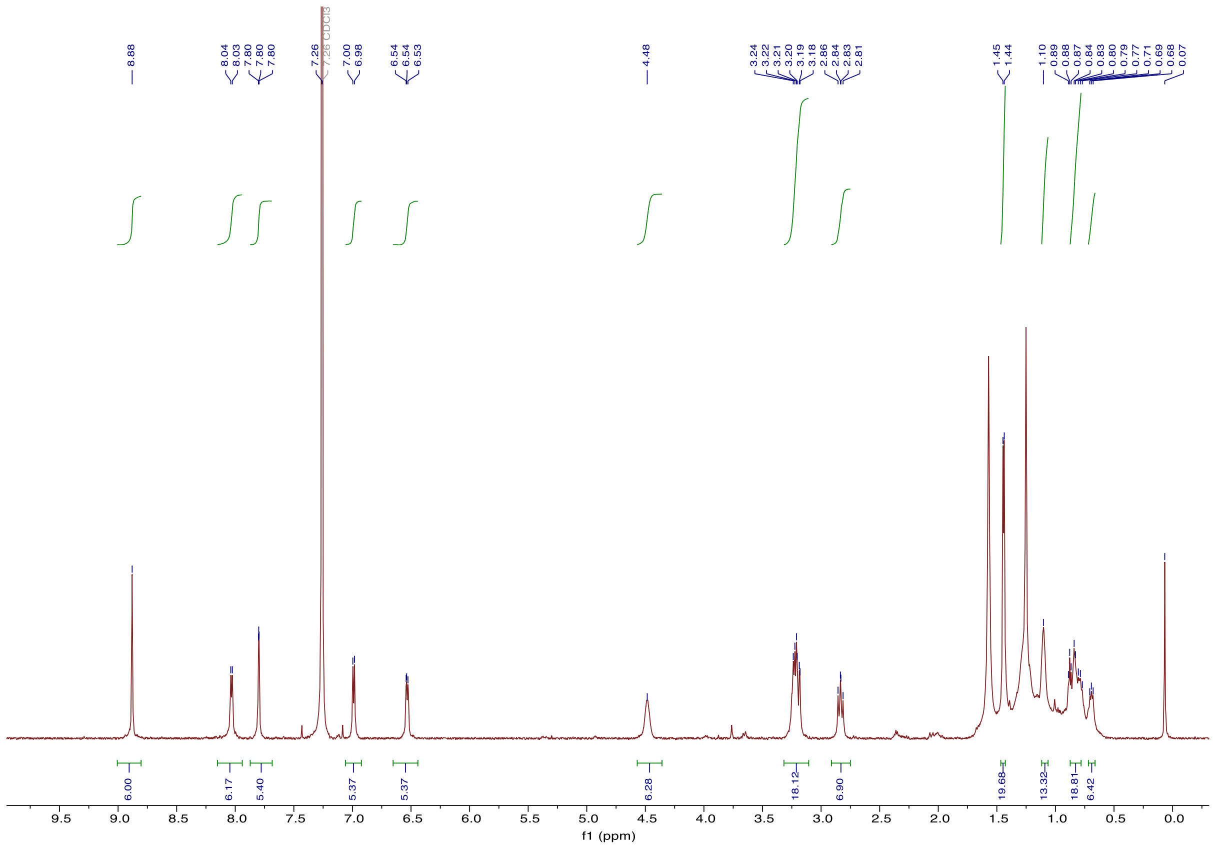

Figure S30: ^{1}H NMR (600 MHz, 298 K, $CDCl_3$) of metal-free trefoil knot Δ-**4**.

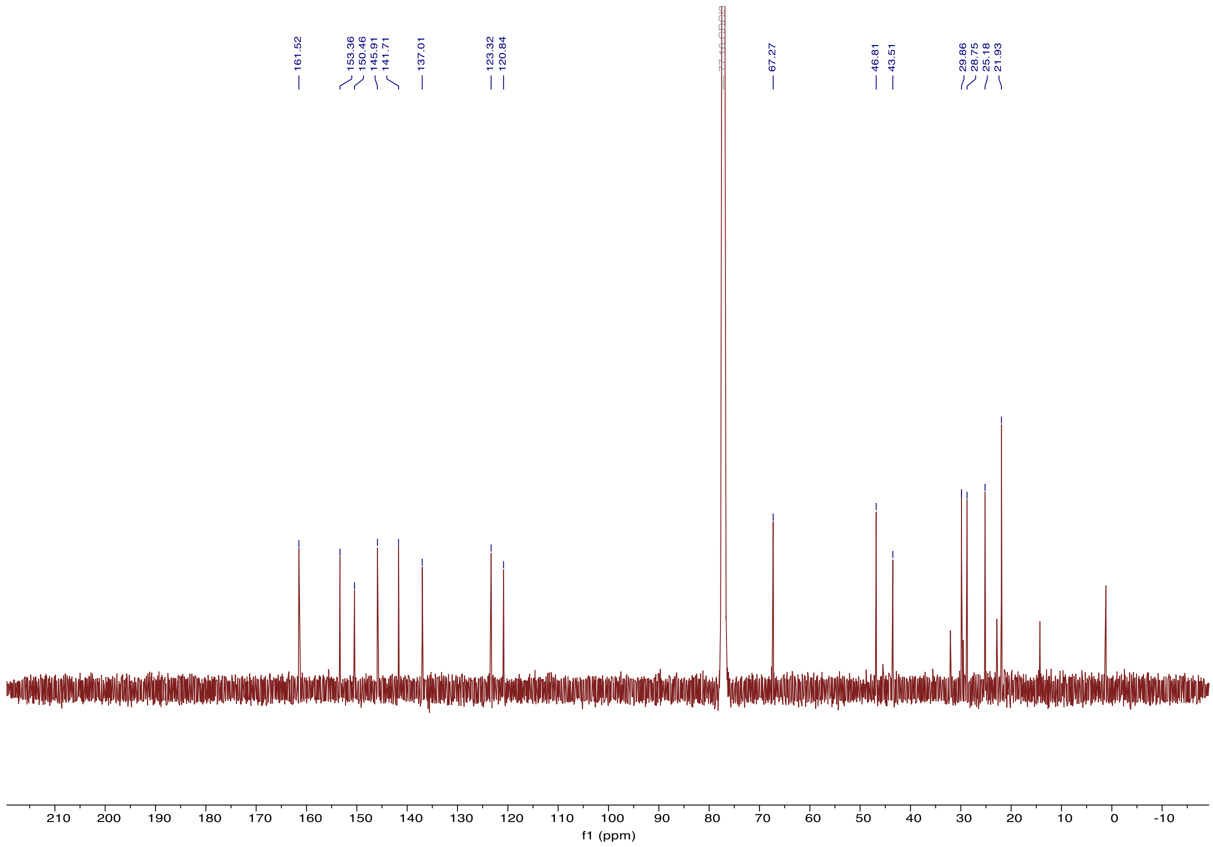

Figure S31: ^{13}C NMR (151 MHz, 298 K, $CDCl_3$) of metal-free trefoil knot Δ-**4**.

7. Molecular Modelling (DFT level) of Trefoil Knot Δ-4

Minimum energy (DFT) structure calculations were performed on Δ-**4** using the Gaussian 09 software package[S7] at the Computational Shared Facility 3 (CSF3) of the University of Manchester. Geometry optimizations were carried out at DFT level using hybrid B3LYP functional with a 6-31G basis set. Chloroform was included as the solvent using the Polarization Continuum Model (PCM). Frequency calculations were performed at the same level to evaluate the zero-point vibrational energy and thermal corrections at 298 K (Table 2).

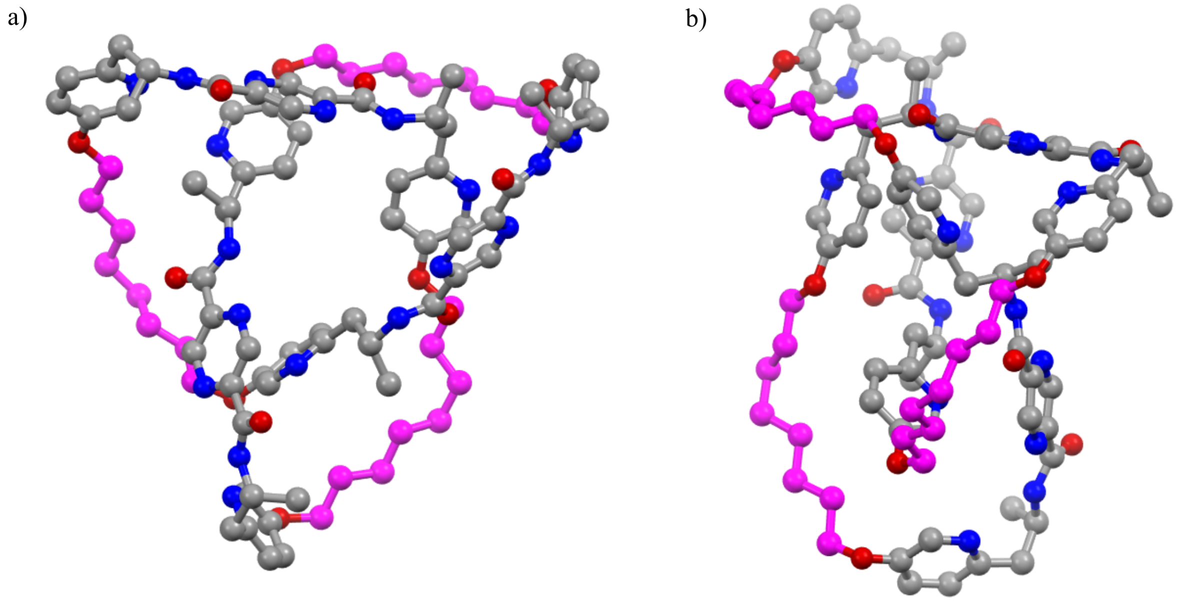

Figure S32: Minimum energy structure (DFT; hybrid B3LYP functional with 6-31G basis set) of the organic trefoil knot Δ-**4** in $CHCl_3$. a) Top view; b) Side-on view. Hydrogen atoms are omitted for clarity. The nitrogen atoms are shown in blue, oxygen atoms in red, and carbon atoms in grey or magenta. For enhanced visibility, the carbon atoms of the hydrocarbon linkers are shown in magenta. The models indicate that π-π and CH-π interactions are abundant in low energy conformations adopted by the metal-free knot, consistent with shielding effects observed in the ^{1}H NMR spectrum of Δ-**4** (Figure 2d).

Table S2. Calculated energy values for trefoil knot **Δ-4**.

**Parameters**	**Energy (Hartree/Particle)**
Zero-point correction	1.989072
Thermal correction to Energy	2.105475
Thermal correction to Enthalpy	2.106419
Thermal correction to Gibbs Free Energy	1.816460
Sum of electronic and zero-point Energies	-5383.071904
Sum of electronic and thermal Energies	-5382.955500
Sum of electronic and thermal Enthalpies	-5382.954556
Sum of electronic and thermal Free Energies	-5383.244515

8. References

S1. Partridge, B. M.; Hartwig, J. F. Brooker, S. *Org. Lett.* **2013***, 15,* 140–143.

S2. Zhong, J.; Zhang, L.; August, D. P.; Whitehead, G. F. S.; Leigh, D. A. *J. Am. Chem. Soc.* **2019**, *141*, 14249-14256.

S3. Gil-Ramírez, G.; Hoekman, S.; Kitching, M. O.; Leigh, D. A.; Vitorica-Yrezabal, I. J.; Zhang, G. *J. Am. Chem. Soc.* **2016**, *138*, 13159–13162.

S4. Nowell, H.; Barnett, S. A.; Christensen, K. E.; Teat, S. J.; Allan, D. R. *J. Synchrotron Radiat.* **2012**, *19*, 435–441.

S5. Rigaku Oxford Diffraction, CrysAlisPro Software system, version 1.171.39.30c, **2017**, Rigaku Corporation, Oxford, UK.

S6. (a) Sheldrick. G. M. *Acta Crystallogr.* **2015**, *C71*, 3–8; (b) Dolomanov, O. V.; Bourhis, L. J.; Gildea, R. J.; Howard, J. A. K.; Puschmann, H. J. *Appl. Crystallogr.* **2009**, *42*, 339–341.

S7. Frisch, M. J.; Trucks, G. W.; Schlegel, H. B.; Scuseria, G. E.; Robb, M. A.; Cheeseman, J. R.; Scalmani, G.; Barone, V.; Mennucci, B.; Petersson, G. A.; Nakatsuji, H.; Caricato, M.; Li, X.; Hratchian, H. P.; Izmaylov, A. F.; Bloino, J.; Zheng, G.; Sonnenberg, J. L.; Hada, M.; Ehara, M.; Toyota, K.; Fukuda, R.; Hasegawa, J.; Ishida, M.; Nakajima, T.; Honda, Y.; Kitao, O.; Nakai, H.; Vreven, T.; Montgomery, J. A., Jr.; Peralta, J. E.; Ogliaro, F.; Bearpark, M.; Heyd, J. J.; Brothers, E.; Kudin, K. N.; Staroverov, V. N.; Kobayashi, R.; Normand, J.; Raghavachari, K.; Rendell, A.; Burant, J. C.; Iyengar, S. S.; Tomasi, J.; Cossi, M.; Rega, N.; Millam, J. M.; Klene, M.; Knox, J. E.; Cross, J. B.; Bakken, V.; Adamo, C.; Jaramillo, J.; Gomperts, R.; Stratmann, R. E.; Yazyev, O.; Austin, A. J.; Cammi, R.; Pomelli, C.; Ochterski, J. W.; Martin, R. L.; Morokuma, K.; Zakrzewski, V. G.; Voth, G. A.; Salvador, P.; Dannenberg, J. J.; Dapprich, S.; Daniels, A. D.; Farkas, Ö.; Foresman, J. B.; Ortiz, J. V.; Cioslowski, J.; Fox, D. J. *Gaussian 09, Revision D.01*, Gaussian, Inc., Wallingford CT, 2013.
